# Circulating sTLR4 and sTREM-1 in Knee Osteoarthritis: Associations with Study-Specific MRI-Defined Categories and Disease Burden

**DOI:** 10.3390/jcm15176902

**Published:** 2026-09-06

**Authors:** Ruhat Ünlü, Hafize Uzun, Naile Mısıroğlu, Bağnu Dündar, Abdulhalim Şenyiğit, Merve Savaş

**Affiliations:** 1 Department of Orthopedics and Traumatology, Istanbul Atlas University, Istanbul 34408, Turkey; 2 Department of Biochemistry, Istanbul Atlas University, Istanbul 34408, Turkey; hafize.uzun@atlas.edu.tr (H.U.); bagnu.dundar@atlas.edu.tr (B.D.); 3 Department of Biochemistry, İstanbul Nişantaşı University, Istanbul 34485, Turkey; nailemisirlioglu@gmail.com; 4 Department of Internal Medicine, Faculty of Medicine, Istanbul Atlas University, Istanbul 34408, Turkey; abdulhalim.senyigit@atlas.edu.tr; 5 Department of Speech and Language Therapy, Istanbul Atlas University, Institute of Health Sciences, Istanbul 34408, Turkey; merve.savas@atlas.edu.tr

**Keywords:** knee osteoarthritis, sTLR4, sTREM-1, MRI-based classification, magnetic resonance imaging, effusion-synovitis, biomarkers, innate immunity

## Abstract

**Background/Objectives:** Osteoarthritis (OA) is biologically heterogeneous, and the relationship between MRI-detected inflammation and circulating innate immune activity remains uncertain. This study aimed to determine whether serum soluble Toll-like receptor 4 (sTLR4) and soluble triggering receptor expressed on myeloid cells-1 (sTREM-1) distinguish between the study-specific MRI-defined INF-OA and non-INF-OA categories, and whether these biomarkers are associated with OA disease burden. **Methods:** This prospective, single-center, cross-sectional study included 30 patients with knee OA and 30 asymptomatic healthy controls. Patients with active OA flare-up, defined by a Knee Osteoarthritis Flare-Ups Score (KOFUS) ≥ 7, were excluded before MRI assessment and category assignment. Patients with OA were assigned to study-specific MRI-defined categories using MRI Osteoarthritis Knee Score (MOAKS) features: INF-OA (*n* = 16) required effusion-synovitis and/or Hoffa-synovitis-related signal alteration graded ≥ 1, whereas non-INF-OA (*n* = 14) required grade 0 for both features. Biomarkers were measured by enzyme-linked immunosorbent assay. Primary within-OA biomarker comparisons were Holm-corrected, and adjusted models included age, sex, and body mass index (BMI). **Results:** Serum sTLR4 and sTREM-1 did not differ between INF-OA and non-INF-OA after Holm correction (adjusted *p* = 0.710 for both). In age-, sex-, and BMI-adjusted models, the INF-OA versus non-INF-OA geometric mean ratios were 1.15 (95% CI 0.92–1.44; *p* = 0.219) for sTLR4 and 1.09 (95% CI 0.89–1.35; *p* = 0.398) for sTREM-1. Given the modest within-OA subgroup sizes, smaller or moderate between-category differences cannot be excluded. After covariate adjustment, the overall OA cohort had higher geometric mean concentrations than controls for sTLR4 (ratio 1.85, 95% confidence interval 1.56–2.20; *p* < 0.001) and sTREM-1 (ratio 1.67, 95% confidence interval 1.43–1.97; *p* < 0.001). None of the systemic inflammatory measurements significantly distinguished INF-OA from non-INF-OA after correction for multiple comparisons. Within the OA cohort, each 1 ng/mL increase in sTLR4 was associated with a 4.99-point higher Western Ontario and McMaster Universities Osteoarthritis Index total score after adjustment for age and body mass index (95% confidence interval 2.28–7.70; *p* < 0.001). **Conclusions:** Circulating sTLR4 and sTREM-1 concentrations were higher in patients with OA than in asymptomatic healthy controls but did not distinguish the study-specific INF-OA and non-INF-OA categories in this sample. These findings should not be interpreted as evidence of biological equivalence between the MRI-defined categories. sTLR4 warrants further evaluation as a circulating correlate of current OA burden in larger longitudinal and multimodal studies.

## 1. Introduction

Osteoarthritis (OA) is a heterogeneous whole-joint disease involving interactions among articular, synovial, subchondral, and periarticular tissues [1]. Patients with similar radiographic disease severity may differ substantially in symptoms, inflammatory activity, structural progression, and treatment response, supporting increasing interest in OA phenotypes and endotypes [1,2]. Phenotypes describe observable clinical or imaging patterns, whereas endotypes refer to underlying molecular or pathobiological mechanisms. Importantly, an imaging-defined phenotype should not be assumed to represent a molecular endotype unless this relationship is supported by biological measurements. Among the proposed OA subgroups, the inflammatory phenotype has attracted particular interest. Synovial inflammation and MRI-detected inflammation-related features, including effusion-synovitis, have been associated with pain, symptom burden, functional impairment, and structural progression [3]. Reliable identification of this phenotype may therefore be clinically relevant because inflammatory OA has been associated with an increased risk of structural progression and subsequent joint replacement and may inform risk-based disease monitoring, patient selection for clinical trials, and the development of phenotype-directed therapeutic strategies [2,4,5].

MRI permits standardized semiquantitative assessment of inflammation-related features by assigning predefined imaging abnormalities to ordinal severity grades. Features such as effusion-synovitis and signal alteration within Hoffa’s fat pad can be evaluated using scoring systems such as the MRI Osteoarthritis Knee Score (MOAKS) [4,6]. However, no universally accepted operational definition of inflammatory OA currently exists. Previous studies have used clinical findings, circulating inflammatory markers, ultrasonography, MRI abnormalities, or combinations of these domains, resulting in substantial heterogeneity in phenotype definitions [7,8]. Consequently, whether locally detected MRI inflammation-related features correspond to a distinct peripheral inflammatory or molecular profile remains uncertain.

A key methodological challenge in phenotype–biomarker studies is circularity: a biomarker used to define a phenotype cannot independently validate that same phenotype. We therefore defined the MRI-based categories independently of circulating inflammatory and biomarker measurements and subsequently tested whether the resulting categories differed in their systemic biological profiles [9,10].

Peripheral blood-derived inflammatory measures, including NLR, PLR, MLR, SII, SIRI, AISI, CRP, and ESR, provide accessible estimates of systemic inflammatory status. Several of these measures have been associated with OA presence or severity, although their associations with disease burden are inconsistent and they are not specific to local joint inflammation [11,12,13,14]. Whether such systemic measures correspond to MRI inflammation-related categories therefore remains uncertain. TLR4 is a pattern-recognition receptor that links tissue-damage signals to innate inflammatory responses and has been implicated in OA pathobiology [15,16,17]. Circulating soluble TLR4 (sTLR4), however, should not be interpreted as a direct measure of membrane-bound TLR4 expression or intra-articular receptor activation. Soluble TLR4 has been detected in OA joint fluid, and circulating sTLR4 has previously been associated with knee OA progression [18,19]. Whether circulating sTLR4 is specific to an MRI-defined inflammatory category or instead reflects broader OA-related disease burden remains uncertain.

Triggering receptor expressed on myeloid cells-1 (TREM-1), expressed predominantly on neutrophils, monocytes, and macrophage-lineage cells, amplifies innate inflammatory responses. Transcriptomic analyses of human OA synovium have demonstrated higher TREM1 expression in relatively inflamed than in less inflamed synovial regions [20], and experimental studies support functional crosstalk between TREM-1- and TLR4-associated signaling [21]. These observations provided the biological rationale for evaluating circulating sTREM-1 as a candidate correlate of inflammatory activity in OA. However, sTREM-1 is not disease-specific: increased circulating concentrations have also been reported in rheumatoid arthritis and in metabolic-risk states such as overweight and obesity [22,23]. Accordingly, sTREM-1 was evaluated in the present study as a candidate systemic correlate rather than as an OA-specific diagnostic biomarker or a direct measure of intra-articular TREM-1 activation.

This study aimed to determine whether the study-specific MRI-defined inflammatory OA category was associated with a distinct systemic inflammatory and circulating innate immune profile. The primary hypothesis was that serum sTLR4 and sTREM-1 concentrations would be higher in the INF-OA category than in the non-INF-OA category. Secondary hypotheses were that the INF-OA category would demonstrate a more pronounced systemic inflammatory profile and that circulating sTLR4 and sTREM-1 concentrations would be higher in patients with OA than in healthy controls. Exploratory analyses examined the associations of sTLR4 and sTREM-1 with systemic inflammatory measurements, symptomatic and radiographic disease burden, and OA disease duration. Their ability to distinguish OA from healthy controls and INF-OA from non-INF-OA was also evaluated on an exploratory, hypothesis-generating basis.

## 2. Materials and Methods

### 2.1. Study Design, Setting, and Reporting Framework

This was a prospective, single-center, cross-sectional observational biomarker study with a healthy comparison group. The study evaluated whether circulating sTLR4 and sTREM-1 were associated with OA disease burden and whether they distinguished the study-specific MRI-defined INF-OA and non-INF-OA categories. Assessment masking was role-specific rather than a formal triple-blind study design. Serum biomarker results were not available during the clinical KOFUS assessment or MRI-based operational category assignment. The MRI reader, radiographic grader, and ELISA personnel were masked to the prespecified information relevant to their respective assessments, as detailed below. This procedural separation was used to minimize the possibility that biomarker or category information influenced clinical, imaging, radiographic, or laboratory measurements. Because this was an observational, non-interventional study without investigator-assigned treatment or other health-related intervention, clinical trial registration was not applicable. This study was conducted at the Istanbul Atlas University Orthopaedics and Traumatology Outpatient Clinic after ethics committee approval had been obtained, and participants were prospectively recruited between November 2025 and May 2026. The report was structured in accordance with the STROBE (Strengthening the Reporting of Observational Studies in Epidemiology) recommendations for observational studies and the STROBE-ME (Strengthening the Reporting of Observational Studies in Epidemiology-Molecular Epidemiology) principles, which particularly emphasize transparent reporting of biological sample collection, processing, storage, laboratory analysis, measurement error, and missing data.

### 2.2. Eligibility and Exclusion Criteria

#### 2.2.1. Participants with OA

Patients were eligible for inclusion in the OA cohort if they were aged ≥ 45 years, presented with symptomatic unilateral knee pain, had knee OA confirmed according to the American College of Rheumatology (ACR) clinical and radiographic classification criteria [24], provided written informed consent, completed the required clinical, laboratory, radiographic, KOFUS, and MRI assessments, and provided a serum sample suitable for biomarker measurement. The index knee was defined as the symptomatic knee and was used consistently for clinical, radiographic, and MRI assessments. Exclusion criteria included rheumatoid arthritis, spondyloarthritis, psoriatic arthritis, crystal arthropathy, septic arthritis, reactive or viral arthritis, other inflammatory arthropathies, systemic autoimmune or inflammatory disease, active systemic infection, malignancy, intra-articular injection involving the index knee within the previous 6 months, and trauma or surgery affecting the index knee within the previous 6 months. Current systemic corticosteroid treatment or immunosuppressive or immunomodulatory therapy was also an exclusion criterion. Metabolic syndrome (MetS) was not a prespecified exclusion criterion and was not formally classified in the present study. According to the harmonized definition, MetS is diagnosed by the presence of any three of five components: increased waist circumference, elevated triglycerides, reduced HDL cholesterol, elevated blood pressure, and elevated fasting glucose or corresponding treatment [25]. Because the complete set of these components was not prospectively collected, retrospective MetS classification was not performed. Body mass index (BMI) was recorded separately as a measure of adiposity and was not considered a surrogate for MetS. Patients with a KOFUS ≥ 7 were considered to have an active OA flare-up, were excluded from the final analytical cohort, and did not proceed to study-specific MRI-based operational category assignment.

#### 2.2.2. Healthy Controls

Healthy controls were eligible if they were aged ≥45 years, had no current knee pain or other knee-related symptoms, provided written informed consent, and had no clinical evidence of knee OA. Control participants were screened for knee OA clinically only; knee radiographs and MRI were not obtained for the purpose of excluding asymptomatic structural OA. Individuals with a history or current evidence of rheumatoid arthritis, spondyloarthritis, psoriatic arthritis, crystal arthropathy, septic arthritis, other inflammatory arthropathies, systemic autoimmune or inflammatory disease, active systemic infection, or malignancy were excluded. Current systemic corticosteroid treatment or immunosuppressive or immunomodulatory therapy was also an exclusion criterion for the control group. Metabolic syndrome was likewise not used as an exclusion criterion for the healthy comparison group and was not retrospectively classified from incomplete metabolic-component data.

### 2.3. Participant Recruitment and Study Population

**Figure 1:**
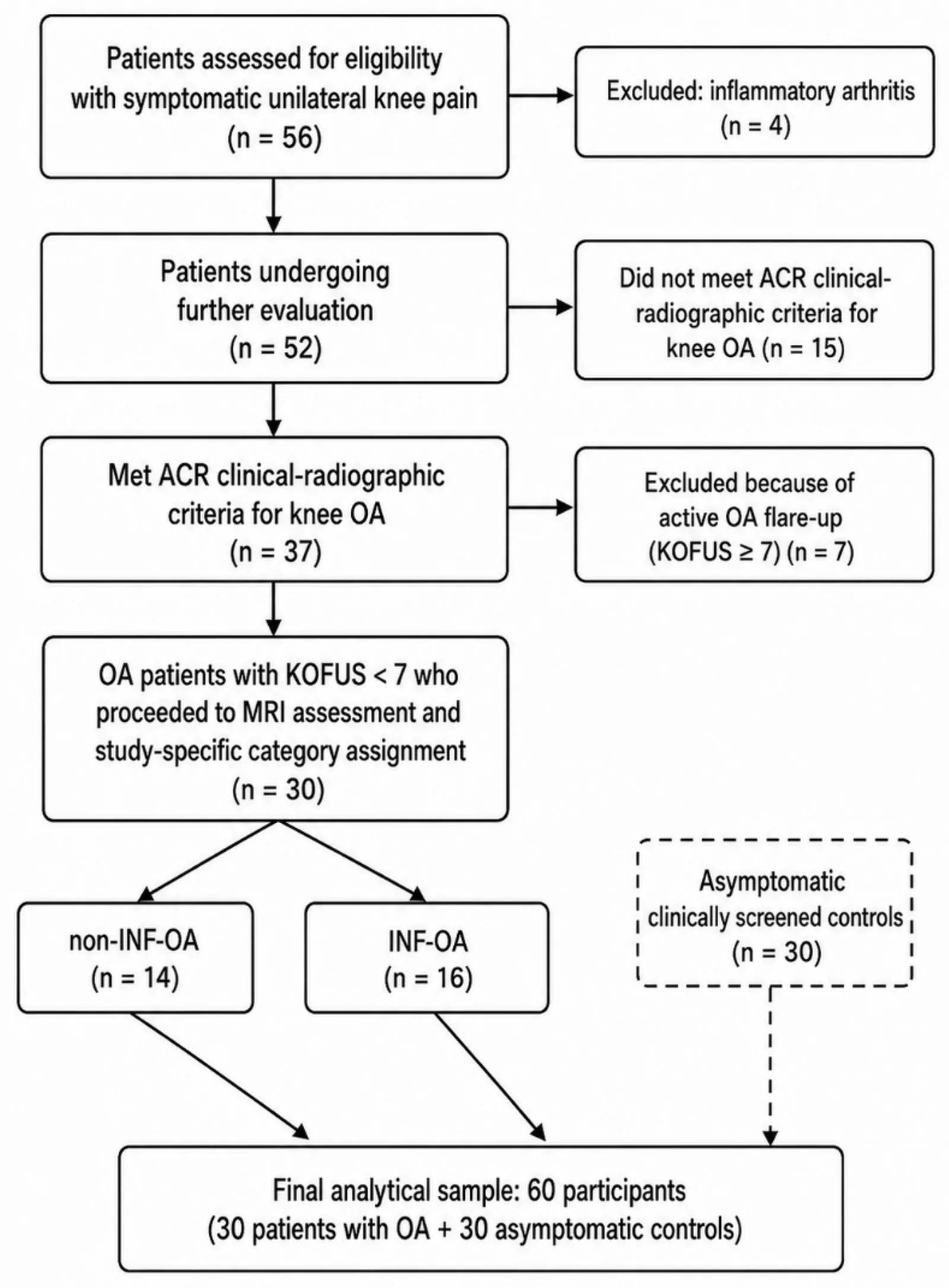
Participant flow from eligibility assessment to the final analytical sample.

Patients presenting with unilateral knee pain were consecutively screened between November 2025 and May 2026. Of 56 patients assessed for eligibility, four were excluded because inflammatory arthritis was identified and 15 did not meet the ACR clinical-radiographic classification criteria for knee OA. Among the 37 patients with confirmed OA, seven were excluded because of active OA flare-up (KOFUS ≥ 7). The remaining 30 patients proceeded to MRI assessment and study-specific category assignment; 16 were assigned to INF-OA and 14 to non-INF-OA. In parallel, 30 asymptomatic healthy controls were recruited from routine health check-up evaluations and selected to have an age and sex distribution comparable to that of the OA cohort. The final analytical sample comprised 60 participants: 30 patients with OA and 30 healthy controls. Participant flow is shown in Figure 1.

### 2.4. Confirmation of the Diagnosis of Knee Osteoarthritis

Knee OA was confirmed according to the ACR clinical and radiographic classification criteria [24]. Participants were required to have knee pain and radiographic evidence of osteophyte formation, together with at least one of the following clinical features: age > 50 years, morning stiffness lasting <30 min, or crepitus on active knee motion. Joint-space narrowing, subchondral sclerosis, and subchondral cyst formation were recorded as supportive radiographic features but were not used as substitutes for the required osteophyte criterion. Patients who did not fulfill these criteria were excluded from the OA cohort.

### 2.5. Exclusion of Inflammatory and Rheumatologic Mimics

Before study-specific MRI-based operational category assignment, potential inflammatory mimics were investigated using a stepwise protocol consisting of clinical screening, first-line laboratory assessment, and disease-specific second-line tests performed only when clinical or laboratory suspicion arose. During the initial evaluation, morning stiffness lasting longer than 60 min, small-joint synovitis, inflammatory back pain, oligoarthritis, psoriasis, nail changes, dactylitis, enthesitis, tophi, acute monoarthritis, fever, constitutional symptoms, recent genitourinary or gastrointestinal infection, Raynaud phenomenon, mucocutaneous findings, and uveitis were assessed.

First-line tests included complete blood count, erythrocyte sedimentation rate (ESR), C-reactive protein (CRP), rheumatoid factor, anti-cyclic citrullinated peptide antibody, antinuclear antibody screening, serum uric acid, complete urinalysis, kidney and liver function tests, and fasting glucose. Disease-specific advanced investigations and validated classification criteria were applied only when considered necessary. Participants in whom inflammatory arthritis, crystal arthropathy, infection, or systemic rheumatologic disease was detected were excluded.

The trigger findings used and the detailed differential diagnostic pathway for rheumatoid arthritis, systemic lupus erythematosus, axial/peripheral spondyloarthritis, psoriatic arthritis, gout, calcium pyrophosphate deposition disease, septic arthritis, reactive arthritis, and viral arthritis are presented in the Supplementary Methods Section [26,27,28,29,30,31,32].

### 2.6. Assessment and Exclusion of Active OA Flare-Up

Active OA flare-up was assessed before study-specific MRI-based operational category assignment using the Knee Osteoarthritis Flare-Ups Score (KOFUS). KOFUS consists of six clinical features: morning stiffness lasting longer than 20 min (1 point), nocturnal awakening due to knee pain (2 points), clinically detected joint effusion (2 points), limping (3 points), knee swelling (3 points), and local warmth (3 points), yielding a total score ranging from 0 to 14 [33]. The KOFUS assessment was performed on the same day as the clinical evaluation and blood sampling and before MRI assessment and subsequent study-specific category assignment. The KOFUS assessment was administered to all patients with confirmed knee OA by a single orthopaedic surgeon using the standardized scoring criteria. At the time of KOFUS assessment, serum sTLR4 and sTREM-1 results were not available to the evaluator, and the study-specific operational MRI category had not yet been assigned. A KOFUS ≥ 7 was considered compatible with active OA flare-up. Patients meeting this threshold were excluded from the final OA analytical cohort and did not proceed to study-specific MRI-based operational category assignment, whereas patients with a KOFUS < 7 proceeded to MRI assessment. The exclusion of patients with a KOFUS ≥ 7 was intended to reduce the influence of clinically overt and potentially transient flare activity on the subsequent comparison between the groups defined by the study-specific MRI classification and circulating biomarkers. KOFUS was used solely to identify and exclude clinically active OA flare-up and was not used to assign the study-specific MRI category. Although KOFUS includes clinically detected joint effusion, swelling, and warmth, these findings represent components of an aggregate clinical flare score rather than imaging-defined synovitis. Importantly, the presence of an individual KOFUS inflammatory feature did not itself result in exclusion; exclusion required a total KOFUS ≥ 7 [33].

### 2.7. Study-Specific Operational MRI-Based Classification

For the purposes of this study, patients with confirmed knee OA were assigned to study-specific operational MRI-based categories after exclusion of inflammatory and rheumatologic mimics and active clinical flare-up (KOFUS < 7). This classification was developed for the present study and was not intended to represent an established or externally validated OA phenotype classification. MRI examinations were performed on the same day as the clinical assessment and blood sampling. All examinations were acquired using the same 3.0-T scanner (SIGNA Pioneer; GE HealthCare, Chicago, IL, USA), a dedicated knee coil, and a standardized non-contrast imaging protocol. The protocol included sagittal, coronal, and axial acquisitions with fat-suppressed proton-density- and T2-weighted sequences and a slice thickness of 3 mm. No intravenous contrast agent was administered. Accordingly, synovial enhancement and enhancing synovial thickness were not directly assessed. The study-specific classification instead relied on the non-contrast MOAKS features of effusion-synovitis and Hoffa-synovitis-related signal alteration as semiquantitative inflammation-related imaging measures. These non-contrast features were not considered reference-standard measurements of synovitis [34,35].

Effusion-synovitis and Hoffa-synovitis were evaluated using the MRI Osteoarthritis Knee Score (MOAKS) system [6]. On non-contrast MRI, effusion-synovitis was interpreted as a composite imaging feature reflecting intra-articular fluid and associated synovial inflammation, whereas Hoffa-synovitis was defined by inflammation-related signal alteration within the infrapatellar fat pad. Both features were graded from 0 to 3, with grade 0 indicating absence, grade 1 mild, grade 2 moderate, and grade 3 severe involvement.

KOFUS screening and MRI assessment served different operational purposes despite partial overlap in inflammation-related findings. KOFUS was used before MRI assessment to identify a clinically active flare using a composite of symptoms and physical examination findings, whereas the subsequent MRI category was assigned using semiquantitative MOAKS imaging features only. Accordingly, clinically detected joint effusion as a KOFUS item and MRI-defined effusion-synovitis were treated as related but non-interchangeable assessments: the former represented a clinical examination finding contributing to the aggregate flare score, whereas the latter represented a semiquantitative imaging feature. Individual KOFUS item scores were not incorporated into the subsequent MRI category assignment [6,33].

**Figure 2:**
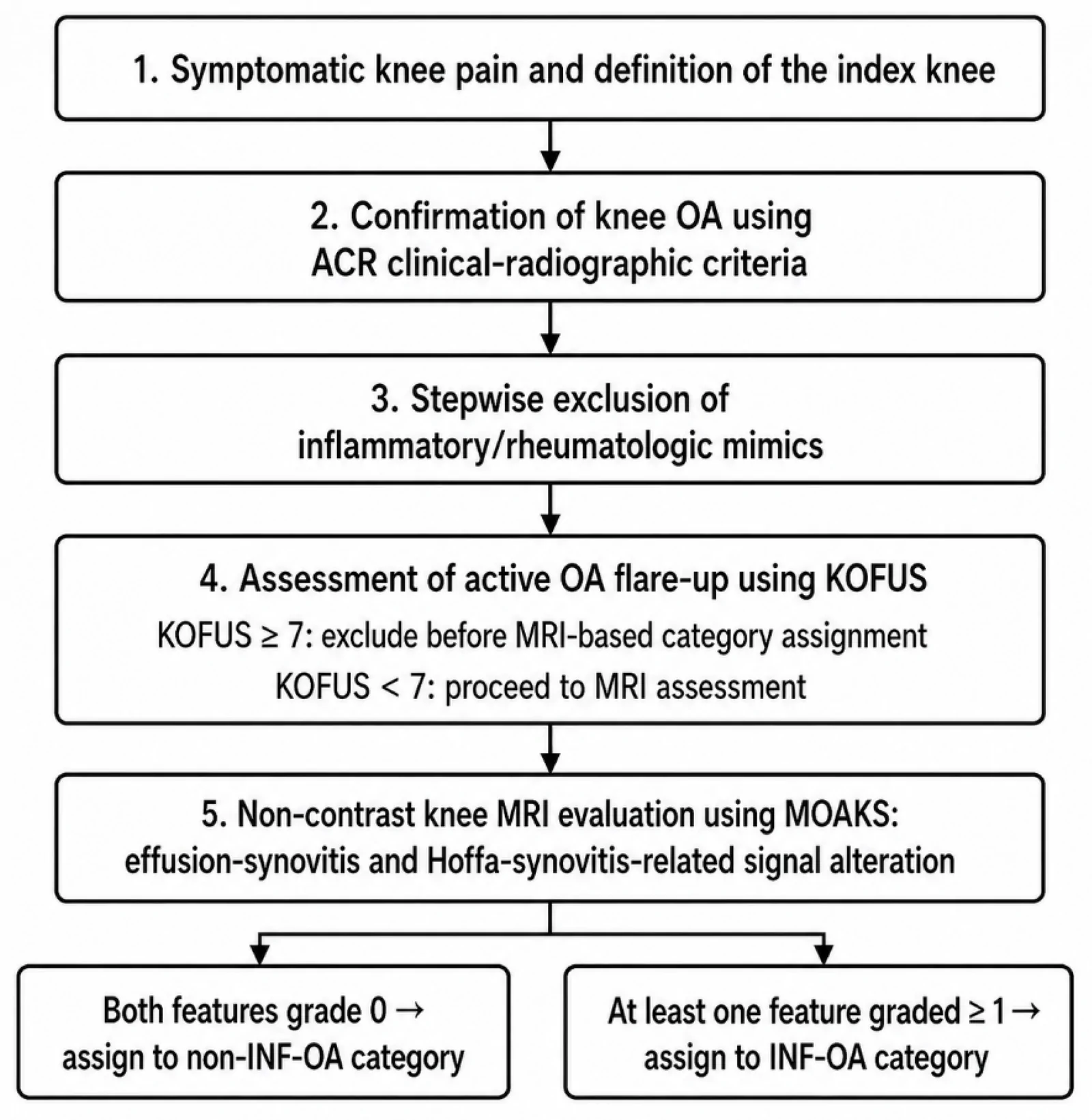
Study-specific operational MRI-based category assignment algorithm. KOFUS was used only to identify active clinical flare before MRI assessment and was not incorporated into operational MRI category assignment. Circulating biomarkers and systemic inflammatory indices were not used for category assignment.

Under this study-specific operational rule, patients with a MOAKS ≥1 for effusion-synovitis and/or Hoffa-synovitis were assigned to the INF-OA category (INF-OA), whereas patients with grade 0 for both features were assigned to the non-INF-OA category. These labels were used solely as operational group definitions within the present study and should not be interpreted as established or externally validated OA phenotype categories. The MOAKS ≥1 threshold was used as a sensitive binary rule to identify the presence of any positive inflammation-related MRI feature and was not intended to represent a severity-based or dose–response classification. Operational category assignment was performed independently of systemic inflammatory and serum biomarker results. NLR, PLR, MLR, SII, SIRI, AISI, CRP, ESR, sTLR4, and sTREM-1 were not used in the classification algorithm. The study-specific operational MRI classification process is illustrated in Figure 2.

### 2.8. MRI Assessment and Reader Blinding

All knee MRI examinations were evaluated by a single radiologist with more than 10 years of experience using the standardized MOAKS scoring criteria. During image evaluation and operational category assignment, the radiologist did not have access to serum sTLR4 or sTREM-1 concentrations, systemic inflammatory indices, CRP, or ESR results.

To assess intra-reader reproducibility, all 30 OA MRI examinations were subsequently re-evaluated by the same radiologist using the standardized MOAKS criteria. Effusion-synovitis and Hoffa-synovitis were rescored independently on their original 0–3 ordinal scales. Intra-reader reliability was quantified using linear weighted Cohen’s kappa and exact percentage agreement. Agreement in the study-specific binary MRI category derived from the repeated MOAKS was additionally evaluated using unweighted Cohen’s kappa.

### 2.9. Clinical and Radiographic Assessments

At baseline, age, sex, body mass index (BMI), current smoking, and alcohol use were recorded. OA-specific clinical variables included disease duration, pain severity, symptom burden, physical function, and radiographic disease severity. Pain severity was assessed using a 10-cm visual analog scale (VAS). OA-related symptoms and physical function were assessed using the validated Turkish version of the Western Ontario and McMaster Universities Osteoarthritis Index (WOMAC) [36], with higher scores indicating greater symptom and functional burden. There were no missing WOMAC data. Weight-bearing anteroposterior and lateral radiographs of the index knee were obtained during the study visit. Radiographic OA severity was evaluated using the Kellgren–Lawrence (KL) grading system by a single orthopaedic surgeon. The orthopaedic surgeon grading the radiographs did not have access to serum biomarker results or the study-specific operational MRI category during Kellgren–Lawrence grading. Higher KL grades indicated more advanced radiographic structural disease severity.

For assessment of intra-reader reproducibility, Kellgren–Lawrence grading was subsequently repeated for all 30 OA radiographs by the same orthopaedic surgeon. Agreement between the original and repeat ordinal KL grades was evaluated using linear weighted Cohen’s kappa and exact percentage agreement.

### 2.10. Routine Laboratory Measurements and Blood Cell-Derived Inflammatory Indices

Routine laboratory assessments were performed using blood samples obtained during the study visit, and all samples were processed on the same day in accordance with the institution’s standard clinical laboratory procedures. Absolute neutrophil, lymphocyte, monocyte, and platelet counts were measured as part of the complete blood count using an automated hematology analyzer (Sysmex XN-1000; Sysmex, Norderstedt, Germany). CRP was measured using an immunoturbidimetric assay on a Cobas c501 analyzer (Roche Diagnostics, Basel, Switzerland) and was reported in mg/L. ESR was determined using the photometric capillary kinetic analysis method on a Test1 analyzer (Alifax, Polverara, Italy) and was reported in mm/h. Blood cell-derived systemic inflammatory indices were calculated using standard formulas described in the literature. Accordingly, the neutrophil-to-lymphocyte ratio (NLR) was calculated as neutrophil count/lymphocyte count, the platelet-to-lymphocyte ratio (PLR) as platelet count/lymphocyte count, the monocyte-to-lymphocyte ratio (MLR) as monocyte count/lymphocyte count, and the aggregate index of systemic inflammation (AISI) as neutrophil count × monocyte count × platelet count/lymphocyte count [37]. The systemic immune-inflammation index (SII) was calculated as platelet count × neutrophil count/lymphocyte count, and the systemic inflammation response index (SIRI) was calculated as neutrophil count × monocyte count/lymphocyte count [38,39]. These indices were calculated after study-specific MRI-based operational category assignment and were not used in INF-OA/non-INF-OA classification. Instead, they were evaluated exclusively as external measures of systemic inflammatory status to characterize the inflammatory profile of the study groups.

### 2.11. Blood Sampling, Serum Processing, and Biomarker Measurements

A total of 5 mL of peripheral venous blood was collected from each participant on the same day as the clinical assessment and KOFUS evaluation under standardized phlebotomy conditions into serum-separator BD Vacutainer^®^ tubes (Becton, Dickinson and Company, Franklin Lakes, NJ, USA). Following collection, the blood samples were allowed to clot for 30 min at room temperature and were subsequently centrifuged at 1500× *g* for 10 min at 4 °C. The resulting serum was carefully separated to minimize contamination with cellular components, aliquoted into single-use cryovials, and stored at −80 °C until biomarker analysis. All samples were processed, aliquoted, and stored using the same preanalytical workflow. Repeated freeze–thaw cycles were avoided, and no sample underwent more than one freeze–thaw cycle before analysis.

A single serum specimen was obtained from each participant and used for both biomarker assays. To improve analytical precision and reduce the influence of technical measurement variability, standards and serum samples were assayed in duplicate wells. Laboratory personnel performing the assays were masked to clinical group, study-specific MRI category, and clinical outcome data.

Serum sTLR4 concentrations were measured using a Human Soluble Toll-like Receptor 4 ELISA Kit (MyBioSource, San Diego, CA, USA; Cat. No. MBS167606). The assay had an analytical measurement range of 0.05–15 ng/mL and a lower limit of detection of 0.022 ng/mL. Serum samples were analyzed without dilution. The manufacturer-reported intra-assay and inter-assay coefficients of variation were <8% and <10%, respectively.

Serum sTREM-1 concentrations were measured using a Human sTREM-1 ELISA Kit (MyBioSource, San Diego, CA, USA; Cat. No. MBS762883). The assay had an analytical measurement range of 31.25–2000 pg/mL and a lower limit of detection of 18.75 pg/mL. In accordance with the manufacturer’s recommendations, serum samples were diluted 1:2 using the supplied sample-dilution buffer, and the measured concentrations were multiplied by the dilution factor to obtain the final concentrations. The manufacturer-reported intra-assay and inter-assay coefficients of variation were 5.30–5.35% and 5.38–5.67%, respectively.

A standard calibration curve was generated for each assay plate using the supplied standards, and sample concentrations were calculated using a four-parameter logistic regression model. Optical density was measured at 450 nm using a Synergy H1 microplate reader (BioTek Instruments, Winooski, VT, USA). All measured concentrations were within the analytical measurement ranges, and no values were below the lower limits of detection.

### 2.12. Study Outcomes and Analytical Hierarchy

The primary within-OA biomarker analyses compared serum sTLR4 and sTREM-1 concentrations between the study-specific INF-OA and non-INF-OA categories. Secondary analyses included comparisons of sTLR4 and sTREM-1 across healthy controls, non-INF-OA, and INF-OA; age-, sex-, and BMI-adjusted group comparisons; comparisons of the overall OA cohort with healthy controls; clinical and radiographic comparisons between the two OA categories; and characterization of systemic inflammatory profiles. Exploratory analyses examined the associations of sTLR4 and sTREM-1 with systemic inflammatory, clinical, and radiographic measures; multivariable associations with symptomatic and radiographic OA burden; and sensitivity models additionally accounting for OA disease duration. ROC analyses and logistic regression models evaluating discrimination between OA and healthy controls or between INF-OA and non-INF-OA were also considered exploratory and hypothesis-generating and were not interpreted as validated diagnostic or classification tools.

### 2.13. Statistical Analysis

Statistical analyses were performed using R version 4.3 and IBM SPSS Statistics version 29.0. All tests were two-sided, and *p* < 0.05 was considered statistically significant. Continuous variables were summarized as mean ± standard deviation or median [interquartile range], according to their distribution, and categorical variables were presented as numbers and percentages. Distributional characteristics were evaluated using graphical inspection and the Shapiro–Wilk test. For two-group comparisons, the independent-samples *t* test was used for approximately normally distributed variables, Welch’s *t* test was used when the homogeneity-of-variance assumption was violated, and the Mann–Whitney U test was used for non-normally distributed variables. For three-group comparisons, one-way analysis of variance followed by Tukey’s honestly significant difference test for post hoc pairwise comparisons was used for approximately normally distributed variables, whereas the Kruskal–Wallis test followed by Dunn’s pairwise comparisons with multiplicity adjustment was used for non-normally distributed variables. Categorical variables were compared using Pearson’s chi-square test or Fisher’s exact test, as appropriate. Effect sizes were reported as Cohen’s d, rank-biserial correlation coefficient (r_rb), epsilon-squared (ε^2^), partial eta-squared (partial η^2^), depending on the analysis.

Holm correction was applied within predefined families of related comparisons. Because sTLR4 and sTREM-1 constituted the two prespecified primary biomarker comparisons between the INF-OA and non-INF-OA categories, Holm correction was applied across these two tests to control the family-wise error rate; both raw and Holm-adjusted *p* values are reported. For direct INF-OA versus non-INF-OA comparisons, NLR, PLR, MLR, SII, SIRI, AISI, CRP, and ESR constituted the systemic inflammatory family and were adjusted together using the Holm procedure. The seven clinical and radiographic outcomes reported in Table 1 were treated as a separate exploratory comparison family and were likewise adjusted using the Holm procedure. Pairwise contrasts from the adjusted three-group biomarker models were corrected within each biomarker using the Holm procedure. In an exploratory sensitivity analysis, MRI inflammation severity was defined as the maximum of the MOAKS effusion-synovitis and Hoffa-synovitis grades for each patient. Participants with INF-OA were stratified as having mild (maximum grade 1) or moderate-to-severe (maximum grade 2–3) inflammation-related MRI findings. Circulating sTLR4 and sTREM-1 concentrations were compared between these severity strata using exact two-sided Mann–Whitney U tests, with rank-biserial correlations reported as effect sizes. Holm correction was applied across the two biomarker comparisons. In addition, Spearman rank correlations were used to explore monotonic associations between the maximum MOAKS inflammation grade (0–3) and each biomarker across the OA cohort, with Holm adjustment across the two correlation tests.

The systemic inflammatory profile was compared across the healthy control, non-INF-OA, and INF-OA groups using Kruskal–Wallis tests. Significant Kruskal–Wallis omnibus effects were followed by Dunn’s pairwise comparisons with multiplicity adjustment. To evaluate systemic inflammatory differences specifically between the study-specific MRI-defined categories, the non-INF-OA and INF-OA groups were also compared directly using Mann–Whitney U tests, with Holm correction applied across the eight systemic inflammatory measurements.

The primary within-OA biomarker analyses compared serum sTLR4 and sTREM-1 concentrations between the non-INF-OA and INF-OA categories using separate unadjusted Mann–Whitney U tests. Secondary unadjusted three-group comparisons among healthy controls, non-INF-OA, and INF-OA were performed using Kruskal–Wallis tests followed by Dunn’s pairwise comparisons with multiplicity adjustment. To account for potential confounding by age, sex, and BMI, separate general linear models were fitted for sTLR4 and sTREM-1. Based on residual diagnostics, biomarker concentrations were log10-transformed. Study group was entered as a fixed factor, and age, sex, and BMI were included as covariates. Estimated marginal means and confidence limits were back-transformed to the original measurement scale and reported as adjusted geometric means and geometric mean ratios with 95% confidence intervals. Overall adjusted group effects were expressed as partial η^2^, and model-based pairwise comparisons were adjusted using the Holm procedure. The log10 transformation improved heteroscedasticity in the sTLR4 model and residual non-normality in the sTREM-1 model. Detailed residual diagnostics, including the Shapiro–Wilk and Breusch–Pagan tests, Cook’s distance, and variance inflation factors, are presented in Supplementary Table S1. To further examine the potential influence of BMI on the biomarker findings, Spearman rank correlations between BMI and circulating sTLR4 and sTREM-1 concentrations were evaluated in the full study sample and separately within the OA cohort. Holm correction was applied across the two biomarker correlations within each cohort. In addition, an exploratory multicategorical indirect-effect analysis was performed to assess whether BMI statistically accounted for group-related differences in biomarker concentrations. Study group was represented by indicator variables with healthy controls as the reference category, BMI was entered as the potential intermediate variable, and log10-transformed sTLR4 or sTREM-1 concentration was the outcome. Age and sex were included as covariates in both the BMI and biomarker models. Indirect effects were estimated for the non-INF-OA versus control, INF-OA versus control, and INF-OA versus non-INF-OA contrasts using 10,000 stratified bootstrap resamples and percentile 95% confidence intervals. Because of the cross-sectional design, these analyses were interpreted as statistical indirect-effect analyses and not as evidence of causal mediation.

In a secondary analysis, the non-INF-OA and INF-OA groups were combined to form the overall OA cohort and were compared with healthy controls. Unadjusted comparisons were performed using the Mann–Whitney U test, whereas adjusted comparisons were conducted using general linear models based on log10-transformed biomarker concentrations and including age, sex, and BMI as covariates. Results were reported as adjusted geometric mean ratios with 95% confidence intervals. Within the OA cohort, associations of sTLR4 and sTREM-1 with systemic inflammatory, clinical, and radiographic variables were evaluated using Spearman rank correlation coefficients. Holm correction was applied separately within the systemic inflammatory and clinical/radiographic outcome families.

The independent association between sTLR4 and symptomatic OA burden was examined using multivariable linear regression with WOMAC total score as the dependent variable. The primary model included sTLR4, age, and BMI. A sensitivity model additionally included OA disease duration. Unstandardized regression coefficients, standardized β coefficients, 95% confidence intervals, and *p* values were reported. Model assumptions were assessed using residual and Q–Q plots and, where appropriate, the Shapiro–Wilk and Breusch–Pagan tests, Cook’s distance, and variance inflation factors. The robustness of the sTLR4 coefficient was further examined using percentile bootstrap confidence intervals based on 10,000 case resamples. To explore potential effect modification of the association between sTLR4 and symptomatic OA burden, three separate interaction models were additionally fitted with WOMAC total score as the dependent variable. Interaction terms between sTLR4 and BMI, age, and the study-specific MRI category (non-INF-OA vs. INF-OA) were examined separately to limit model overparameterization in the 30-patient OA cohort. Continuous predictors were mean-centered before construction of the interaction terms. Each model retained the corresponding lower-order main effects, and age and BMI were included as covariates as appropriate. Holm correction was applied across the three interaction tests. These analyses were considered exploratory.

The association between sTLR4 and radiographic OA severity was assessed using a proportional-odds ordinal logistic regression model with Kellgren–Lawrence grade as the ordinal outcome. The primary model included sTLR4, age, and BMI, and a sensitivity model additionally included OA disease duration. The proportional-odds assumption was evaluated using the test of parallel lines. The effect of sTLR4 was reported as an odds ratio both per 1 ng/mL increase and per 1-standard-deviation increase within the OA cohort. Percentile bootstrap confidence intervals based on 10,000 case resamples were calculated to assess coefficient robustness. To examine whether the sparse distribution of Kellgren–Lawrence grades influenced the ordinal regression estimates, two additional exploratory sensitivity analyses were performed. First, radiographic severity was dichotomized as mild (KL grade 2) versus moderate-to-severe (KL grades 3–4), and the association with sTLR4 was evaluated using binary logistic regression. Second, within the moderate-to-severe subgroup, KL grade 4 was compared with KL grade 3 using a separate binary logistic regression model. Because the analytic OA cohort contained no KL grade 1 cases, the mild stratum comprised only KL grade 2 and therefore did not permit a within-stratum ordinal regression. Given the small subgroup sizes, particularly the five patients with KL grade 4, these sensitivity models were intentionally parsimonious and unadjusted. Holm correction was applied across the two sensitivity tests.

Exploratory discrimination of OA from healthy controls was evaluated separately for sTLR4 and sTREM-1 using receiver operating characteristic (ROC) curve analyses. Areas under the curve (AUCs) and their 95% confidence intervals were estimated using 10,000 stratified bootstrap resamples. To assess internal predictive optimism, leave-one-out cross-validation (LOOCV) was additionally performed using separate single-biomarker logistic regression models. In each of 60 leave-one-out iterations, one participant was held out, the model was fitted to the remaining 59 participants, and an out-of-fold predicted probability was generated for the omitted participant. LOOCV AUCs were then calculated from the pooled out-of-fold predictions. Optimal sample-specific biomarker thresholds were identified by maximizing the Youden index (J = sensitivity + specificity − 1), and the corresponding sensitivity, specificity, and Youden J values were reported. These thresholds were derived from the full sample and were considered exploratory; they were not interpreted as validated diagnostic or screening cutoffs in the absence of independent external validation.

The exploratory ability of sTLR4 and sTREM-1 to distinguish INF-OA from non-INF-OA was evaluated using single-biomarker binary logistic regression models and a combined two-biomarker model. ROC analyses were conducted for each biomarker and for the predicted probabilities obtained from the combined model, and AUCs were reported with 95% confidence intervals. Owing to the limited number of patients in the study-specific MRI-defined categories, these models were kept parsimonious and did not include additional covariates. Their results were interpreted as hypothesis-generating rather than as validated prediction or classification performance.

The approved study protocol specified a target sample of 60 participants, comprising 30 patients with knee OA and 30 healthy controls, for the planned OA-versus-control biomarker comparison. As specified in the revised analytical hierarchy, the direct INF-OA versus non-INF-OA biomarker comparison constituted the primary within-OA analysis, whereas the OA-versus-healthy-control comparison was a secondary analysis. The archived protocol documented an anticipated effect-size parameter of 0.5, a significance level of α = 0.05, statistical power of 80% (1 − β = 0.80; β = 0.20), equal allocation between the OA and control groups (1:1), and calculation using G*Power (version 3.1.9.7 (Heinrich Heine University Düsseldorf, Düsseldorf, Germany)). Because the original protocol documentation did not retain the specific effect-size metric or G*Power test module, these historical parameters were not retrospectively reinterpreted. To provide a reproducible assessment of the statistical sensitivity of the available sample, an additional two-sided independent-groups sensitivity analysis was performed using G*Power, with α = 0.05 and 80% power. With 30 participants per group, the OA-versus-control design had a minimum detectable standardized mean difference of d = 0.736. No separate a priori sample-size calculation was performed for the study-specific INF-OA versus non-INF-OA comparison. For the obtained subgroup sizes of 14 non-INF-OA and 16 INF-OA participants, the corresponding sensitivity analysis yielded a minimum detectable standardized mean difference of d = 1.062 at α = 0.05 and 80% power. Accordingly, the subgroup comparison was sufficiently sensitive to detect only relatively large standardized between-category differences, whereas smaller or moderate differences could not be reliably excluded. There were no missing clinical, laboratory, imaging, or biomarker data in the final analytical sample; therefore, no imputation was performed.

### 2.14. Ethical Approval and Informed Consent

The study was conducted in accordance with the principles of the Declaration of Helsinki. The research protocol was deemed medically and ethically appropriate by the Istanbul Atlas University Clinical Research Ethics Committee (Reference No.: E-22686390-050.99-80213; Decision Date: 14 October 2025; Decision No.: 11/01). Written informed consent was obtained from all participants before study inclusion and before biological sampling procedures were performed. Participants’ clinical data and biological samples were de-identified using unique study codes, and analyses were performed using de-identified data.

## 3. Results

### 3.1. Participant Characteristics and OA Categories

The final analytical sample comprised 60 participants: 30 healthy controls, 14 patients with non-INF-OA, and 16 patients with INF-OA. The groups were comparable in age, sex distribution, current smoking, and alcohol use. An overall group effect was observed for body mass index (BMI) (F(2,57) = 3.24, *p* = 0.046); however, none of the Tukey-adjusted pairwise comparisons reached statistical significance (control vs. non-INF-OA, adjusted *p* = 0.091; control vs. INF-OA, adjusted *p* = 0.117; non-INF-OA vs. INF-OA, adjusted *p* = 0.981) (Table 2).

**Table 1:** OA-specific clinical and radiographic characteristics according to study-specific MRI-defined category.

Variable	non-INF-OA (*n* = 14)	INF-OA (*n* = 16)	Effect Size	Raw *p*	Holm-Adjusted *p*
OA disease duration, years	7.93 ± 3.27	5.81 ± 3.51	d = 0.62	0.100	0.403
VAS pain score	6.57 ± 1.20	7.25 ± 1.26	d = 0.55	0.143	0.430
WOMAC pain	13.86 ± 3.70	14.31 ± 3.59	d = 0.13	0.735	0.735
WOMAC stiffness	4.0 [2.0]	5.0 [2.0]	r_rb = 0.241	0.260	0.521
WOMAC function	36.07 ± 9.91	44.69 ± 6.55	d = 1.04	0.008	0.058
WOMAC total	54.07 ± 13.86	63.81 ± 7.56	d = 0.87	0.030	0.180
Kellgren–Lawrence grade	2.5 [1.0]	3.0 [0.0]	r_rb = 0.344	0.081	0.403

Data are presented as mean ± standard deviation or median [IQR], as appropriate. Normally distributed continuous variables were compared using Student’s independent-samples *t*-test. Welch’s *t*-test was used for WOMAC total because the homogeneity-of-variance assumption was violated. WOMAC stiffness and Kellgren–Lawrence grade were compared using the Mann–Whitney U test. Effect sizes are reported as absolute Cohen’s d or rank-biserial correlation (r_rb), as appropriate. To account for multiple clinical and radiographic comparisons between the two OA categories, Holm correction was applied across the seven outcomes reported in this table. Both raw and Holm-adjusted *p* values are presented. All tests were two-sided.

**Table 2:** Demographic and baseline clinical characteristics of the study groups.

Variable	Control (*n* = 30)	non-INF-OA (*n* = 14)	INF-OA (*n* = 16)	*p* Value
Age, years	58.1 ± 7.49	60.9 ± 6.29	62.9 ± 7.67	0.101
BMI, kg/m^2^	27.0 ± 4.58	30.1 ± 3.90	29.8 ± 5.01	0.046 †
Female sex, *n* (%)	15 (50.0)	7 (50.0)	8 (50.0)	1.000
Current smoking, *n* (%)	9 (30.0)	5 (35.7)	4 (25.0)	0.873
Alcohol use, *n* (%)	3 (10.0)	1 (7.1)	4 (25.0)	0.360

Data are presented as mean ± standard deviation or *n* (%), as appropriate. Continuous variables were compared using one-way analysis of variance (ANOVA), and categorical variables were compared using Pearson’s chi-square test or Fisher’s exact test, as appropriate. When the overall ANOVA was statistically significant, post hoc pairwise comparisons were performed using Tukey’s honestly significant difference test with adjustment for multiple comparisons. † For BMI, the overall group effect was statistically significant (*p* = 0.046); however, none of the Tukey-adjusted pairwise comparisons reached statistical significance: control vs. non-INF-OA, adjusted *p* = 0.091; control vs. INF-OA, adjusted *p* = 0.117; and non-INF-OA vs. INF-OA, adjusted *p* = 0.981.

No clinical or radiographic outcome differed significantly between the two study-specific OA categories after Holm correction, although nominal differences were observed for WOMAC function and total scores (Table 1).

Intra-reader reproducibility was high across the imaging assessments. Exact agreement for MOAKS effusion-synovitis was 96.7% (linear weighted κ = 0.967), and agreement for Hoffa-synovitis was 86.7% (linear weighted κ = 0.854). Exact agreement for Kellgren–Lawrence grading was 86.7% (linear weighted κ = 0.793). Repeated application of the study-specific MRI classification rule using the repeat MOAKS yielded complete agreement for the INF-OA/non-INF-OA category (100% agreement; Cohen’s κ = 1.000).

### 3.2. Systemic Inflammatory Profile

All eight systemic inflammatory measures differed significantly across the control, non-INF-OA, and INF-OA groups (all *p* < 0.001), with effect sizes ranging from ε^2^ = 0.390 for PLR to ε^2^ = 0.699 for AISI. These differences were driven by higher values in both OA groups than in healthy controls. Although ESR was higher in INF-OA than in non-INF-OA in the three-group post hoc analysis (multiplicity-adjusted within-marker *p* = 0.030), direct comparison of the two study-specific OA categories showed that none of the eight systemic inflammatory measures remained statistically significant after Holm correction across the full eight-marker family. The nominal ESR difference (raw *p* = 0.012) did not remain significant after family-wise adjustment (Holm-adjusted *p* = 0.096) (Table 3 and Supplementary Table S2).

### 3.3. Circulating sTLR4 and sTREM-1

In the primary within-OA biomarker comparisons, neither circulating sTLR4 nor sTREM-1 differed significantly between the non-INF-OA and INF-OA groups (sTLR4: raw *p* = 0.355, Holm-adjusted *p* = 0.710; sTREM-1: raw *p* = 0.603, Holm-adjusted *p* = 0.710) (Table 4, Panel A). In the exploratory severity-stratified analysis, 6 of the 16 patients with INF-OA had a maximum MOAKS inflammation grade of 1 and 10 had a maximum grade of 2–3. sTLR4 concentrations did not differ significantly between patients with grade 1 and grade 2–3 findings (median [IQR], 5.76 [0.69] vs. 5.98 [1.65] ng/mL; U = 27.0; r_rb = 0.10; raw *p* = 0.792; Holm-adjusted *p* = 0.792). Likewise, sTREM-1 concentrations did not differ significantly between the two severity strata (median [IQR], 505.2 [71.6] vs. 449.7 [183.6] pg/mL; U = 39.0; r_rb = −0.30; raw *p* = 0.368; Holm-adjusted *p* = 0.735). Across the entire OA cohort, maximum MOAKS inflammation grade was not significantly associated with either sTLR4 (Spearman ρ = 0.206; raw *p* = 0.275; Holm-adjusted *p* = 0.551) or sTREM-1 (ρ = 0.023; raw *p* = 0.905; Holm-adjusted *p* = 0.905) (Supplementary Table S3).

After adjustment for age, sex, and BMI, the overall group effect remained significant for both log10-transformed sTLR4 (F(2,54) = 26.76, *p* < 0.001, partial η^2^ = 0.498) and sTREM-1 (F(2,54) = 21.06, *p* < 0.001, partial η^2^ = 0.438). Both OA groups had significantly higher adjusted concentrations than controls, whereas the adjusted INF-OA versus non-INF-OA contrasts were not statistically significant for either biomarker. BMI was not independently associated with log10-sTLR4 (*p* = 0.093) or log10-sTREM-1 (*p* = 0.703) (Table 4, Panels B and C). Consistently, BMI was not significantly correlated with either biomarker in the full sample (sTLR4: Spearman ρ = −0.020, *p* = 0.878; sTREM-1: ρ = 0.232, *p* = 0.075) or within the OA cohort (sTLR4: ρ = −0.318, *p* = 0.087; sTREM-1: ρ = 0.125, *p* = 0.510). After Holm correction across the two biomarker correlations within each cohort, none of these associations remained statistically significant. In exploratory indirect-effect analyses, none of the bootstrap 95% confidence intervals for the BMI-related indirect effects excluded zero for either sTLR4 or sTREM-1, providing no statistical evidence that BMI accounted for the observed between-group biomarker differences (Supplementary Table S4).

**Table 4:** Primary and adjusted circulating sTLR4 and sTREM-1 findings. Panel (**A**). Primary within-OA biomarker comparisons; Panel (**B**). Age-, sex-, and BMI-adjusted three-group models. Panel (**C**). Holm-adjusted model contrasts.

**(A)**														
**Biomarker**			**non-INF-OA**		**INF-OA**			**U**		**r_rb**	**Raw *p***	**Holm-Adjusted *p***		
sTLR4, ng/mL			5.39 [1.95]		5.92 [1.21]			89.0		0.205	0.355	0.710		
sTREM-1, pg/mL			490.95 [133.45]		489.20 [152.28]			99.0		0.116	0.603	0.710		
**(B)**														
**Biomarker**	**Adjusted Group Effect**			**Partial η^2^**		**Control Adjusted Geometric Mean (95% CI)**			**non-INF-OA Adjusted Geometric Mean (95% CI)**				**INF-OA Adjusted Geometric Mean (95% CI)**	
sTLR4, ng/mL	F(2,54) = 26.76; *p* < 0.001			0.498		2.99 (2.66–3.36)			5.15 (4.36–6.08)				5.92 (5.06–6.92)	
sTREM-1, pg/mL	F(2,54) = 21.06; *p* < 0.001			0.438		284.71 (255.29–317.53)			454.92 (389.30–531.61)				497.51 (429.27–576.59)	
**(C)**														
**Biomarker**		**Comparison**					**Geometric Mean Ratio (95% CI)**							**Adjusted *p***
sTLR4		non-INF-OA vs. control					1.72 (1.40–2.12)							<0.001
sTLR4		INF-OA vs. control					1.98 (1.62–2.42)							<0.001
sTLR4		INF-OA vs. non-INF-OA					1.15 (0.92–1.44)							0.219
sTREM-1		non-INF-OA vs. control					1.60 (1.32–1.94)							<0.001
sTREM-1		INF-OA vs. control					1.75 (1.45–2.11)							<0.001
sTREM-1		INF-OA vs. non-INF-OA					1.09 (0.89–1.35)							0.398

Panel (**A**) values are presented as median [interquartile range]. The two prespecified primary within-OA comparisons, one for sTLR4 and one for sTREM-1, were treated as a single multiplicity family, and Holm correction was applied across these two tests; both raw and Holm-adjusted *p* values are reported. Adjusted analyses in Panels (**B**,**C**) used log10-transformed biomarker concentrations, and estimated marginal means were back-transformed to the original scale. Pairwise model contrasts in Panel (**C**) were adjusted using the Holm procedure. All tests were two-sided.

When the non-INF-OA and INF-OA categories were combined, adjusted geometric mean concentrations remained higher in OA than in controls for sTLR4 (geometric mean ratio 1.85, 95% CI 1.56–2.20; *p* < 0.001) and sTREM-1 (geometric mean ratio 1.67, 95% CI 1.43–1.97; *p* < 0.001). Exploratory OA-control ROC analyses yielded apparent AUCs of 0.875 for sTLR4 and 0.877 for sTREM-1; the corresponding LOOCV AUCs were 0.846 and 0.850, respectively. The sample-specific Youden thresholds were ≥4.51 ng/mL for sTLR4 and ≥385.2 pg/mL for sTREM-1, each yielding 83.3% sensitivity and 83.3% specificity (Youden J = 0.667) (Supplementary Table S5). These estimates should not be interpreted as validated diagnostic or screening performance.

Exploratory discrimination analyses between the study-specific MRI-defined categories showed that neither sTLR4 (OR = 1.28, 95% CI 0.76–2.17; *p* = 0.357) nor sTREM-1 (OR = 1.003, 95% CI 0.996–1.010; *p* = 0.446) was significantly associated with INF-OA category membership. The combined two-biomarker model was also not significant (likelihood-ratio χ^2^(2) = 1.40; *p* = 0.497). Consistently, ROC analyses showed limited discrimination between INF-OA and non-INF-OA for sTLR4 (AUC = 0.603, 95% CI 0.385–0.821; *p* = 0.356), sTREM-1 (AUC = 0.442, 95% CI 0.228–0.656; *p* = 0.596), and the combined model-derived probability (AUC = 0.612, 95% CI 0.395–0.828; *p* = 0.312). Full results are provided in Supplementary Table S6. Complete distributional characteristics of sTLR4 and sTREM-1, including mean, standard deviation, variance, median, interquartile range, and minimum–maximum values, are presented in Supplementary Table S7.

### 3.4. Clinical and Radiographic Associations

Within the OA cohort, higher sTLR4 concentrations remained significantly associated after Holm correction with higher WOMAC total score (ρ = 0.669), worse WOMAC function (ρ = 0.569), greater WOMAC stiffness (ρ = 0.538), higher Kellgren–Lawrence grade (ρ = 0.672), and higher ESR (ρ = 0.591). sTREM-1 remained significantly associated only with WOMAC stiffness (ρ = 0.534). Neither biomarker was associated with OA disease duration. The complete correlation matrix is provided in Supplementary Table S8.

In the age- and BMI-adjusted linear regression model, each 1 ng/mL increase in sTLR4 was associated with a 4.99-point higher WOMAC total score (95% CI 2.28–7.70; *p* < 0.001). The association remained significant after additional adjustment for OA disease duration (B = 4.73, 95% CI 1.90–7.57; *p* = 0.002). The primary sTLR4 coefficient was stable in 10,000 bootstrap resamples (bootstrap 95% CI 2.31–7.46) (Table 5, Panel A; Supplementary Table S9). Exploratory interaction analyses provided no statistically significant evidence that the association between sTLR4 and WOMAC total score was modified by BMI (sTLR4 × BMI: B = 0.101, 95% CI −0.664 to 0.866; raw *p* = 0.788; Holm-adjusted *p* = 1.000), age (sTLR4 × age: B = −0.042, 95% CI −0.581 to 0.497; raw *p* = 0.873; Holm-adjusted *p* = 1.000), or the study-specific MRI category (sTLR4 × MRI category: B = −3.853, 95% CI −8.821 to 1.115; raw *p* = 0.122; Holm-adjusted *p* = 0.367) (Supplementary Table S10).

In the proportional-odds model adjusted for age and BMI, each 1-standard-deviation increase in sTLR4 (1 SD = 1.448 ng/mL) was associated with greater odds of belonging to a higher Kellgren–Lawrence category (OR = 17.18, 95% CI 2.93–100.60; *p* = 0.002). The association remained significant after additional adjustment for OA disease duration (OR = 15.30, 95% CI 2.83–82.71; *p* = 0.002) (Table 5, Panel B). However, the bootstrap distribution for the ordinal model was markedly right-skewed, and only 9396 of 10,000 resamples yielded finite estimates; the model-based Wald confidence interval was therefore retained as the primary interval, and the effect magnitude should be interpreted cautiously (Supplementary Table S9). The KL-grade distribution in the OA cohort was 9 patients with grade 2, 16 with grade 3, and 5 with grade 4; no patient had KL grade 1. Median [IQR] sTLR4 concentrations increased across these categories (KL2: 4.24 [3.08–5.20] ng/mL; KL3: 5.85 [5.40–6.35] ng/mL; KL4: 7.60 [6.69–7.66] ng/mL). In the dichotomized sensitivity analysis, each 1 ng/mL increase in sTLR4 was associated with greater odds of belonging to the KL3–4 group rather than the KL2 group (OR = 6.21, 95% CI 1.47–26.27; raw *p* = 0.013; Holm-adjusted *p* = 0.026). Within the moderate-to-severe subgroup, the association remained directionally consistent for KL4 versus KL3 (OR = 3.62 per 1 ng/mL, 95% CI 1.01–12.91; raw *p* = 0.047; Holm-adjusted *p* = 0.047). Although these sensitivity analyses preserved the direction of association, the confidence intervals remained wide, consistent with limited precision arising from the small grade-specific sample sizes (Supplementary Table S11).

**Table 5:** Adjusted associations of circulating sTLR4 with clinical and radiographic OA severity. Panel (**A**). Multiple linear regression models for WOMAC total score; Panel (**B**). Proportional-odds ordinal logistic regression models for Kellgren–Lawrence grade.

**(A)**								
**Model**	**Predictor**	**B**		**95% CI**		**Standardized β**		***p* Value**
Primary	sTLR4, ng/mL	4.99		2.28–7.70		0.611		<0.001
	Age, years	−0.25		−0.78 to 0.28		−0.147		0.340
	BMI, kg/m^2^	−0.02		−0.91 to 0.86		−0.008		0.959
Sensitivity	sTLR4, ng/mL	4.73		1.90–7.57		0.579		0.002
	Age, years	−0.26		−0.80 to 0.27		−0.156		0.319
	BMI, kg/m^2^	−0.12		−1.05 to 0.81		−0.045		0.795
	OA disease duration, years	−0.41		−1.53 to 0.72		−0.120		0.466
**(B)**								
**Model**	**Predictor**		**OR**		**95% CI**		***p* Value**	
Primary	sTLR4, per 1-SD increase		17.18		2.93–100.60		0.002	
	Age, years		0.90		0.79–1.03		0.121	
	BMI, kg/m^2^		1.02		0.83–1.25		0.850	
Sensitivity	sTLR4, per 1-SD increase		15.30		2.83–82.71		0.002	
	Age, years		0.90		0.78–1.02		0.103	
	BMI, kg/m^2^		0.96		0.77–1.20		0.725	
	OA disease duration, years		0.79		0.57–1.10		0.158	

Primary models were adjusted for age and BMI; sensitivity models additionally included OA disease duration. One standard deviation of sTLR4 in the OA cohort was 1.448 ng/mL. Alternative scaling and bootstrap sensitivity results are presented in Supplementary Table S9.

## 4. Discussion

### 4.1. Principal Findings

This study evaluated circulating sTLR4 and sTREM-1 in knee OA at three conceptually distinct levels: association with the study-specific MRI-defined inflammatory category, broader differences between patients with OA and asymptomatic healthy controls, and associations with current symptomatic and radiographic disease burden. Three principal findings emerged. First, the primary within-OA biomarker hypothesis was not supported: neither sTLR4 nor sTREM-1 differed significantly between the study-specific INF-OA and non-INF-OA categories. Adjusted between-category contrasts were likewise not statistically significant, and exploratory single- and two-biomarker models showed limited discrimination between the MRI-defined categories. Second, both OA categories demonstrated higher systemic inflammatory measurements and higher circulating sTLR4 and sTREM-1 concentrations than asymptomatic healthy controls. These OA-control differences persisted for both biomarkers after adjustment for age, sex, and BMI. Third, within the OA cohort, sTLR4 showed consistent associations with WOMAC-defined symptomatic and functional burden and with radiographic severity, whereas the clinical associations of sTREM-1 were more limited.

Collectively, these findings suggest that the study-specific MRI-defined inflammatory category was not closely reflected by the circulating innate immune measurements evaluated in this sample. Although sTLR4, sTREM-1, and systemic inflammatory measurements were elevated in OA overall, none showed a reproducible profile specific to the INF-OA category. Thus, sTLR4 and sTREM-1 appear more consistent with candidate circulating correlates of broader OA-related biological activity than with circulating markers specific to the study-specific MRI category.

The negative between-category comparisons should not, however, be interpreted as evidence that INF-OA and non-INF-OA are biologically equivalent. The INF-OA and non-INF-OA categories comprised only 16 and 14 patients, respectively, and no separate a priori sample-size calculation was performed for this comparison. Sensitivity analysis indicated that these subgroup sizes provided 80% power at α = 0.05 only for a standardized between-category difference of approximately d = 1.06 or greater. Consistent with this limited precision, the adjusted INF-OA versus non-INF-OA geometric mean ratios had 95% confidence intervals of 0.92–1.44 for sTLR4 and 0.89–1.35 for sTREM-1, indicating that smaller or moderate between-category differences cannot be excluded despite the nonsignificant comparisons. This statistical caution is also consistent with the broader OA literature, which indicates that MRI-detected inflammation, synovial tissue inflammation, and circulating inflammatory measurements represent related but non-interchangeable biological domains. Peripheral inflammatory activity does not necessarily parallel concurrent MRI-defined inflammation, while tissue-level inflammatory heterogeneity may show only limited correspondence with circulating measurements [40,41,42]. Accordingly, the present data provide no positive evidence that circulating sTLR4 or sTREM-1 distinguishes the study-specific operational MRI categories, but they do not establish biological equivalence between INF-OA and non-INF-OA.

The clinical comparisons between the two study-specific MRI categories showed a partially different pattern. The INF-OA group showed higher WOMAC function and total scores at the nominal level, with relatively large effect sizes (d = 1.04 and d = 0.87, respectively); however, these differences did not remain statistically significant after Holm correction (adjusted *p* = 0.058 and 0.180, respectively). No other clinical or radiographic comparison differed significantly between the two study-specific OA categories. Accordingly, the observed WOMAC differences should be interpreted as exploratory signals rather than as evidence of clinically distinct symptom burden between the operational MRI categories.

### 4.2. MRI-Defined Inflammation and Systemic Biological Profiles

MRI-detected inflammation, histological synovitis, synovial-fluid mediators, and circulating inflammatory measures reflect related but non-interchangeable domains of OA biology. In the present study, systemic inflammatory measures were elevated in OA overall but did not distinguish the study-specific INF-OA and non-INF-OA categories after family-wise correction; the nominal ESR difference likewise did not survive Holm adjustment. This pattern is consistent with evidence showing limited correspondence between circulating hsCRP and MRI-detected knee inflammation [40], whereas tissue-based studies demonstrate substantial synovial inflammatory heterogeneity that may relate to structural disease burden without providing reliable systemic classification of individual patients [41,42]. Together, these findings indicate that local imaging, synovial tissue, and peripheral blood measures should be interpreted as complementary rather than interchangeable markers of inflammation. The limited sTLR4 literature similarly spans different biological compartments and outcome definitions, further emphasizing that circulating and intra-articular measurements should not be assumed to represent interchangeable dimensions of OA inflammation [18,19].

Several features of the study-specific operational MRI classification may also have reduced between-category contrast. Using a MOAKS grade ≥ 1 for either effusion-synovitis or Hoffa-synovitis-related signal alteration grouped mild grade 1 abnormalities with more pronounced grade 2 or 3 findings and combined two imaging features that may not represent identical underlying tissue processes. In addition, non-contrast MRI cannot directly assess synovial enhancement and cannot distinguish synovial thickening from joint fluid as directly as contrast-enhanced MRI. Comparative studies have shown that Hoffa fat-pad signal alteration on non-enhanced MRI is a sensitive but non-specific surrogate of synovitis, whereas effusion-synovitis shows better correspondence with synovial thickening assessed on gadolinium-enhanced MRI [34,35]. These features should therefore be interpreted as semiquantitative inflammation-related MRI indicators rather than direct or reference-standard measurements of synovitis [6,33]. Excluding patients with a KOFUS ≥ 7 reduced the likelihood that an active clinical flare would influence the operational MRI classification, but it may also have removed patients with the most overt acute inflammatory activity and thereby attenuated biological differences between the remaining categories. Accordingly, the present findings are most applicable to patients with symptomatic knee OA who were not experiencing a clinically active flare at the time of assessment.

Overall, the elevation of peripheral inflammatory measures in both OA categories supports a broader systemic inflammatory milieu accompanying symptomatic OA but does not establish local MRI-category specificity. Because indices such as NLR, PLR, MLR, SII, SIRI, and AISI are influenced by systemic immune, metabolic, and comorbidity-related factors, they should not be interpreted as stand-alone indicators of local knee inflammation or as substitutes for imaging-based classification.

### 4.3. Interpretation of sTLR4

The sTLR4 findings indicate a stronger relationship with OA and current disease burden than with the study-specific MRI-defined category. Circulating sTLR4 was higher in both OA categories than in healthy controls, and the adjusted comparison of the combined OA cohort with controls remained significant after accounting for age, sex, and BMI. However, sTLR4 did not differ significantly between the INF-OA and non-INF-OA categories and showed limited exploratory discrimination between the two categories. Thus, the higher sTLR4 concentrations observed in OA cannot be interpreted as evidence specific to the study-specific MRI-defined inflammatory category.

Previous studies support the relevance of soluble TLR4-related biomarkers in OA but have focused primarily on their presence across biological compartments and their relationship with disease progression rather than concurrent symptom burden. Soluble TLRs, including sTLR4, have been detected in OA joint fluid [18]. In the DOXY trial cohort of 431 patients with symptomatic knee OA, baseline and time-integrated plasma sTLR4 were associated with longitudinal change in urinary C-terminal telopeptide of type II collagen (CTX-II), supporting a relationship between circulating TLR4-related biology and OA disease activity or progression [19]. The present study addresses a complementary question by linking serum sTLR4 cross-sectionally to contemporaneous WOMAC-defined symptomatic and functional burden in patients with established knee OA. Direct comparison with the limited previous human sTLR4 literature highlights important methodological differences. Barreto et al. (2017) examined sTLR4 locally in synovial fluid, including 10 patients with knee OA, 10 healthy knee controls, and 10 patients with first carpometacarpal OA; no MRI-based inflammatory classification was used [18]. Synovial-fluid sTLR4 concentrations were higher in knee OA than in healthy knees, providing evidence that soluble TLR4 is detectable and altered within the OA joint environment. Huang et al. (2018) [19], in contrast, evaluated circulating sTLR4 longitudinally in 431 participants from the DOXY trial. That cohort consisted of obese women aged 45–64 years with unilateral radiographic knee OA, defined by KL grade 2 or 3 in the index knee and KL grade 0 or 1 in the contralateral knee, without MRI-based inflammatory classification [19]. Plasma sTLR4 was measured longitudinally and was associated primarily with changes in the biochemical cartilage-degradation marker uCTX-II rather than with the MRI-defined inflammatory category or concurrent symptomatic burden examined in the present study. Thus, the current study differs from both previous reports by evaluating serum sTLR4 in relation to the study-specific MRI inflammation-related category while simultaneously examining contemporaneous WOMAC-defined symptoms and radiographic burden. A side-by-side comparison of these studies is provided in Supplementary Table S12.

Within the OA cohort, sTLR4 remained associated after Holm correction with WOMAC stiffness, function, and total scores, KL grade, and ESR. Each 1 ng/mL higher serum sTLR4 concentration was associated with a 4.99-point higher WOMAC total score after adjustment for age and BMI (95% CI 2.28–7.70), and the estimate remained similar after additional adjustment for OA disease duration. On the 0–96 WOMAC total scale used in this study, a 4.99-point difference corresponds to approximately 5.2% of the full scale. Expressed over one standard deviation of sTLR4 in the OA cohort (1.448 ng/mL), the fitted coefficient corresponds to an approximately 7.2-point difference in WOMAC total score. These values provide clinical scale context for the magnitude of the association but should not be interpreted as an established minimal clinically important difference. Published WOMAC minimal important change and difference estimates vary according to population, intervention, scoring scale, and estimation method. In a systematic review of knee OA outcome measures after non-surgical interventions, the median minimal important difference (MID) for WOMAC total was approximately 6.8 points on a 0–100 scale, while the corresponding ROC-based estimate was 7.8 points; these values correspond to approximately 6.5 and 7.5 points, respectively, on the 0–96 scale used in the present study [43]. Accordingly, the 4.99-point fitted difference associated with a 1 ng/mL higher sTLR4 concentration is below these published WOMAC-total MID benchmarks, corresponding to approximately 67–76% of their magnitude. When expressed over one standard deviation of sTLR4 in the OA cohort (1.448 ng/mL), however, the fitted difference is approximately 7.2 WOMAC points, which approaches these published MID estimates. Nevertheless, the present coefficient represents a cross-sectional between-person association rather than within-patient symptom change over time. Accordingly, this comparison is intended only to contextualize the magnitude of the association and does not imply that a 1 ng/mL change in sTLR4 would produce a five-point change in WOMAC symptoms or that the observed association itself represents a clinically important change.

Nevertheless, the cross-sectional design prevents determination of directionality. Higher circulating sTLR4 could accompany greater tissue damage and inflammatory activity, participate in regulatory responses to ongoing innate immune stimulation, or reflect systemic processes associated with more symptomatic disease. Soluble TLR4 should not be regarded as a direct measurement of membrane-bound TLR4 expression, receptor signaling intensity, or intra-articular activation of the MyD88-, TRIF-, NF-κB-, or IRF3-related pathways. The present findings therefore support the interpretation of sTLR4 as a candidate circulating correlate of current OA burden rather than as direct mechanistic evidence of increased joint TLR4 signaling.

The association between sTLR4 and radiographic severity was directionally consistent with the correlation findings and persisted after adjustment for age, BMI, and disease duration. However, the estimated odds ratio from the proportional-odds model was large, its confidence interval was wide, and the bootstrap distribution was markedly right-skewed, with a proportion of resamples failing to yield finite estimates. These features indicate instability arising from the small sample and sparse distribution across Kellgren–Lawrence categories. Consequently, the direction of the radiographic association is noteworthy, but its estimated magnitude should not be considered definitive. The additional KL-severity sensitivity analyses supported this interpretation. The positive association with sTLR4 persisted after collapsing the sparse ordinal categories into KL2 versus KL3–4 and remained directionally consistent when KL4 was compared with KL3 within the moderate-to-severe subgroup. Thus, the radiographic association does not appear to be attributable solely to the small number of KL4 observations. Nevertheless, confidence intervals remained wide, and the absence of KL1 cases together with only five KL4 cases substantially limited grade-specific precision. These analyses provide additional support for the direction, but not the magnitude, of the observed radiographic association. The WOMAC total model provides substantially more stable support for an association between sTLR4 and current disease burden than the ordinal radiographic model does.

### 4.4. Interpretation of sTREM-1

Circulating sTREM-1 was also higher in both OA categories than in asymptomatic healthy controls, and this difference persisted in the adjusted OA-control analysis. However, sTREM-1 did not differ significantly between the INF-OA and non-INF-OA categories and showed limited exploratory discrimination between the two categories, either alone or in combination with sTLR4. Within the OA cohort, only its association with WOMAC stiffness remained significant after correction for multiple comparisons. Associations with broader functional, total symptom, and radiographic outcomes did not survive multiplicity adjustment.

The magnitude of circulating sTREM-1 elevation should be interpreted in the context of its known lack of disease specificity. In the present study, adjusted sTREM-1 concentrations were approximately 1.67-fold higher in the overall OA cohort than in healthy controls. In rheumatoid arthritis, Choi et al. reported plasma concentrations of 170.10 ± 84.71 pg/mL in patients with RA compared with 97.41 ± 40.64 pg/mL in healthy controls, corresponding to an approximately 1.75-fold difference; concentrations were further increased in clinically active RA [22]. In a metabolic-risk population, Cifci et al. reported serum sTREM-1 concentrations of 407.3 ± 323.7 pg/mL in overweight adults compared with 150.3 ± 152.7 pg/mL in normal-weight controls, an approximately 2.7-fold difference [23]. Absolute concentrations should not be compared directly across studies because specimen matrix, assay platform, calibration, population characteristics, and inflammatory context differ. Nevertheless, these observations demonstrate that elevations of a magnitude comparable to, or greater than, that observed in the present OA cohort occur in non-OA inflammatory and metabolic conditions. Reported sTREM-1 thresholds are likewise highly context-dependent and are not transferable across diseases. For example, a baseline threshold of >390 pg/mL was evaluated for predicting methotrexate response in rheumatoid arthritis but showed limited performance (61.5% sensitivity and 59.3% specificity), whereas a serum threshold of ≥133 pg/mL yielded 71.1% sensitivity and 73.3% specificity for distinguishing sepsis from systemic inflammatory response syndrome [44,45]. These markedly different thresholds further illustrate that no universal or OA-specific circulating sTREM-1 cutoff has been established.

Local tissue studies support a role for TREM-1-related biology in OA but do not imply that serum sTREM-1 should parallel MRI inflammation. Transcriptomic analysis of inflamed and relatively non-inflamed regions of synovial tissue obtained from the same patients with OA identified differential expression of inflammatory genes, including marked upregulation of TREM1 in inflamed regions [20]. Experimental macrophage studies have also demonstrated functional interaction between TREM-1 and TLR4-associated responses, with TREM-1 contributing to amplification of innate immune signaling [21]. However, local synovial TREM1 expression, membrane TREM-1 signaling, and circulating soluble TREM-1 concentrations are biologically distinct measurements.

The elevation of sTREM-1 in the overall OA cohort may therefore reflect a broader myeloid inflammatory environment accompanying OA rather than a profile specific to the study-specific MRI category. The isolated association with WOMAC stiffness is difficult to assign to a single mechanism because stiffness may arise from synovial and capsular changes, altered joint-fluid characteristics, structural restriction, muscle dysfunction, and periarticular processes. This finding should be treated as exploratory and requires independent replication rather than a specific mechanistic interpretation.

The contrasting clinical association profiles of sTLR4 and sTREM-1 also indicate that the two soluble receptors are not interchangeable indicators of a single linear pathway. Although cellular TLR4 and TREM-1 signaling may interact, their circulating soluble forms may be generated, regulated, cleared, and compartmentalized differently. The present results suggest that circulating sTLR4 and sTREM-1 may capture partly different aspects of damage sensing and myeloid inflammatory activity.

### 4.5. Clinical and Research Implications

The current findings do not support using sTLR4, sTREM-1, or routinely derived systemic inflammatory indices as stand-alone tests for assignment to the study-specific MRI-defined INF-OA/non-INF-OA categories. A biomarker may be elevated in OA overall without distinguishing between the study-specific MRI-defined categories, and these two interpretations should not be conflated.

The exploratory OA-control ROC analyses produced relatively high apparent AUC estimates for both biomarkers. Internal validation using LOOCV yielded somewhat lower but similar AUCs (0.846 for sTLR4 and 0.850 for sTREM-1), indicating modest attenuation relative to the apparent estimates. However, these analyses compared patients with OA with asymptomatic, clinically screened healthy controls rather than with a diagnostically challenging clinical population comprising patients with other causes of knee pain. Moreover, LOOCV provides only internal validation within the same small dataset, and no independent external validation was performed. Accordingly, the sample-specific Youden thresholds and associated sensitivity and specificity estimates should be interpreted as exploratory separation of two contrasting groups rather than as clinically applicable screening or diagnostic cutoffs. The present design also does not establish OA specificity, because the biomarkers were not evaluated against clinically relevant comparator groups with other causes of knee pain, other musculoskeletal disorders, or inflammatory conditions. Demonstration of diagnostic value or disease specificity would require independent external validation in larger and clinically heterogeneous cohorts.

The association of sTLR4 with WOMAC-defined burden supports its further evaluation in larger studies, but not its immediate clinical use. Longitudinal cohorts should determine whether changes in sTLR4 parallel changes in pain and function and whether baseline concentrations are associated with subsequent structural or symptomatic trajectories. Such studies should incorporate repeated biomarker measurements because OA endotypes may not remain constant over time. In the IMI-APPROACH cohort, biomarker-based endotypes showed only moderate longitudinal stability, with average overall stability of approximately 55% and inflammatory-endotype stability of approximately 54% [46]. More recent cytokine profiling from the same programme showed substantial temporal fluctuation and limited endotype discrimination across a panel of 15 circulating cytokines, further illustrating the challenges of inferring a stable molecular state from a single blood measurement [47].

Future endotyping studies should therefore integrate complementary rather than interchangeable biological domains. Same-day peripheral blood, synovial fluid, contrast-enhanced or quantitative imaging, and, where ethically and clinically feasible, synovial tissue measurements would help determine whether circulating sTLR4 and sTREM-1 relate to local receptor expression, inflammatory-cell composition, synovial mediator concentrations, or structural tissue damage. Repeated assessments would additionally clarify whether MRI and circulating biomarker patterns are stable traits or time-dependent states. Such multimodal and longitudinal designs may be more informative than attempts to define an inflammatory endotype using a single imaging feature or soluble biomarker.

### 4.6. Strengths and Limitations

A methodological strength was the separation of MRI category assignment from circulating biomarker and systemic inflammatory measurements.

Additional strengths included prospective consecutive screening, structured exclusion of inflammatory and rheumatologic mimics, and exclusion of patients experiencing an active OA flare before MRI-based category assignment. Clinical assessment, blood sampling, and MRI were performed on the same day, reducing temporal mismatch between local imaging and circulating measurements. MRI examinations were acquired using the same 3.0-T scanner and standardized protocol and were evaluated by an experienced radiologist blinded to biomarker results. Serum samples underwent a standardized preanalytical workflow, ELISA measurements were performed in duplicate by personnel blinded to clinical group and MRI category, and all measured concentrations were within the analytical ranges. Analytically, adjustment for age, sex, and BMI, correction for multiple comparisons within predefined outcome families, disease-duration sensitivity models, and bootstrap analyses strengthened the assessment of robustness. Exploratory interaction analyses likewise did not provide statistically significant evidence that the association between sTLR4 and WOMAC-defined symptom burden varied according to age, BMI, or the study-specific MRI category. However, given the small OA cohort, particularly after stratification by MRI category, these analyses had limited power to detect modest effect modification and should be interpreted cautiously.

Several limitations should also be acknowledged. First, the study was conducted at a single center and included only 30 patients with OA, divided into relatively small INF-OA and non-INF-OA groups. The between-category comparisons were therefore underpowered to exclude small or moderate differences, and the findings may not generalize to broader populations, other OA stages, or patients experiencing an active flare. However, the exclusion of patients with a KOFUS ≥ 7 also introduced spectrum restriction by excluding patients with the greatest clinically apparent inflammatory activity and may therefore have attenuated biological and biomarker contrasts between the groups defined by the study-specific MRI classification. Accordingly, the findings should not be generalized to patients experiencing an active OA flare. No separate a priori sample-size calculation was performed for the primary INF-OA versus non-INF-OA comparison.

Second, the cross-sectional design prevents causal or prognostic interpretation. The observed biomarker associations cannot determine whether higher sTLR4 or sTREM-1 contributes to OA burden, results from OA-related tissue damage, or reflects correlated systemic processes. In particular, the present findings cannot establish that sTLR4 predicts pain progression, functional decline, MRI changes, or radiographic progression.

Third, the study-specific MRI classification was based on non-contrast MRI. Accordingly, synovial enhancement was not directly assessed, and effusion-synovitis and Hoffa-synovitis-related signal alteration should be considered semiquantitative surrogate measures rather than reference-standard assessments of synovitis [34,35]. The operational MOAKS ≥ 1 rule was designed to identify any positive inflammation-related MRI feature but may have reduced between-category contrast by combining mild grade 1 abnormalities with more pronounced grade 2 or 3 findings and by combining effusion-synovitis with Hoffa fat-pad signal alteration.

However, the additional exploratory severity-stratified analysis did not show higher circulating sTLR4 or sTREM-1 concentrations in patients with grade 2–3 findings compared with those with grade 1 findings, and neither biomarker showed a significant monotonic association with maximum MOAKS inflammation grade. These exploratory analyses did not provide evidence that inclusion of mild grade 1 abnormalities substantially diluted the primary between-category biomarker comparison. Nevertheless, the small numbers within the severity strata limit precision, and modest severity-dependent associations cannot be excluded.

Fourth, MRI examinations were assessed by a single radiologist, and radiographic grading was performed by a single orthopaedic surgeon. Although repeat evaluation demonstrated high intra-reader reproducibility for MOAKS effusion-synovitis, Hoffa-synovitis, Kellgren–Lawrence grading, and the derived INF-OA/non-INF-OA classification, inter-reader reliability could not be assessed and remains a limitation of the study. Masking was implemented through role-specific restriction of access to biomarker, imaging-category, and clinical outcome information. However, no independent blinding-integrity questionnaire, formal masking audit, or dedicated blinding-quality-control record was collected; therefore, adherence to masking cannot be independently validated beyond the documented procedural separation of assessments.

Fifth, the study did not include synovial fluid or synovial tissue measurements. Consequently, the relationships between circulating soluble receptors, local soluble receptor concentrations, membrane receptor expression, synovial cellular composition, and tissue-level inflammatory signaling could not be evaluated. Accordingly, the present data cannot directly determine the correspondence between local and circulating inflammatory biology or establish a local–systemic biological dissociation.

Sixth, the healthy comparison group represented an asymptomatic, clinically screened reference group rather than a real-world differential-diagnosis population. In addition, control participants did not undergo knee radiography or MRI; therefore, asymptomatic structural OA cannot be excluded. The control group should consequently be interpreted as an asymptomatic, clinically screened reference group rather than an imaging-confirmed structurally normal population, which limits interpretation of the OA-control biomarker and discrimination analyses. Formal MetS status was not available because all components required for harmonized MetS classification were not prospectively collected. Although BMI was included in the adjusted biomarker and disease-burden models, BMI does not fully capture the MetS construct; therefore, residual metabolic confounding cannot be excluded. Additional analyses did not demonstrate significant correlations between BMI and either circulating biomarker, and the exploratory indirect-effect analyses provided no statistical evidence that BMI accounted for the observed group differences in sTLR4 or sTREM-1. However, because the present data are cross-sectional, these analyses cannot establish causal mediation or temporal ordering. Nevertheless, the independent association between MetS and knee OA appears to be substantially attenuated after adjustment for BMI or body weight in the available literature, and MetS has not consistently been associated with structural progression in established knee OA [48,49,50]. Residual confounding from medication exposure, unmeasured comorbidity, or other systemic inflammatory determinants also remains possible. Accordingly, the OA-control differences and ROC estimates should not be generalized to clinical diagnostic settings.

Finally, the proportional-odds model for KL grade produced a wide confidence interval and unstable bootstrap distribution. Although the direction of association was consistent with the correlation analysis and disease-duration sensitivity model, the magnitude of the radiographic association requires replication in a substantially larger sample with adequate representation across radiographic grades.

## 5. Conclusions

Among patients with knee OA without an active clinical flare, circulating sTLR4 and sTREM-1 concentrations were higher than in asymptomatic healthy controls but did not distinguish the study-specific operational INF-OA and non-INF-OA categories. Given the modest within-OA subgroup sizes, smaller or moderate between-category biomarker differences cannot be excluded. Systemic inflammatory measurements were likewise elevated in OA overall but did not show a reproducible pattern specific to the study-specific INF-OA category after multiplicity correction. Within this sample and under the operational MRI definition used, these findings suggest limited correspondence between MRI-detected inflammation-related features and circulating inflammatory measures; they should not be interpreted as establishing a general biological dissociation between local and systemic inflammation in OA.

Within the OA cohort, higher sTLR4 concentrations were associated with greater WOMAC-defined symptomatic and functional burden, whereas the association with radiographic severity was directionally consistent but statistically unstable. sTREM-1 showed more limited clinical associations. These exploratory findings support further evaluation of sTLR4 as a candidate circulating correlate of current OA burden in larger longitudinal and multimodal studies but do not establish diagnostic, MRI-category classification, molecular-endotype, or prognostic utility. These findings should not be extrapolated to patients experiencing an active OA flare.

## Figures and Tables

**Table 3:** Systemic inflammatory profiles across the control, non-inflammatory OA, and inflammatory OA groups.

Variable	Control	non-INF-OA	INF-OA	χ^2^	ε^2^	*p* Value	Significant Pairwise Comparisons
NLR	1.55 [0.50]	2.94 [1.05]	3.11 [1.11]	32.1	0.544	<0.001	Control < both OA groups
PLR	106.95 [42.60]	188.17 [38.50]	166.35 [60.50]	23.0	0.390	<0.001	Control < both OA groups
MLR	0.17 [0.11]	0.31 [0.16]	0.36 [0.21]	29.7	0.503	<0.001	Control < both OA groups
SII	374.74 [203.00]	878.67 [342.00]	849.39 [394.00]	31.8	0.538	<0.001	Control < both OA groups
SIRI	0.60 [0.44]	1.59 [0.64]	1.92 [0.83]	38.4	0.650	<0.001	Control < both OA groups
AISI	162.26 [87.50]	469.82 [276.00]	534.99 [250.00]	41.2	0.699	<0.001	Control < both OA groups
CRP	2.05 [1.97]	6.70 [4.93]	6.60 [5.42]	29.9	0.507	<0.001	Control < both OA groups
ESR	11.50 [5.75]	27.50 [18.00]	34.00 [13.00]	38.1	0.645	<0.001	Control < both OA groups; non-INF-OA < INF-OA, adjusted *p* = 0.030

Data are presented as median [interquartile range]. Overall differences were evaluated using Kruskal–Wallis tests, with epsilon-squared (ε^2^) as the effect size. Significant omnibus tests were followed by multiplicity-adjusted nonparametric pairwise comparisons. The ESR difference between the two study-specific OA categories did not remain significant after Holm correction across the full eight-marker panel (Supplementary Table S2).

## Data Availability

The de-identified numerical data supporting the findings of this study are available from the corresponding author upon reasonable request, subject to institutional and ethical approval. The data are not publicly available because of participant privacy, data-protection requirements, and institutional restrictions.

## Supplementary Materials

The following supporting information can be downloaded at: https://www.mdpi.com/article/10.3390/jcm15176902/s1, Supplementary Methods S1: Trigger-Based Exclusion of Inflammatory and Rheumatologic Mimics; Table S1: Transformation rationale and diagnostic checks; Table S2: Systemic inflammatory profiles according to study-specific MRI-defined category; Table S3: Exploratory analyses of circulating sTLR4 and sTREM-1 according to MOAKS inflammation severity; Table S4: Associations of BMI with circulating biomarkers and exploratory BMI indirect-effect analyses; Table S5: Unadjusted three-group and All-OA versus healthy control biomarker analyses; Table S6: Exploratory discrimination between the study-specific MRI-defined OA categories; Table S7: Distributional Characteristics of Serum sTLR4 and sTREM-1; Table S8: Associations of circulating sTLR4 and sTREM-1 with systemic inflammatory, clinical, and radiographic measures among patients with OA; Table S9: Alternative scaling and bootstrap sensitivity analyses for sTLR4; Table S10: Exploratory interaction analyses of the association between circulating sTLR4 and WOMAC total score; Table S11: Sensitivity analyses of the association between circulating sTLR4 and Kellgren–Lawrence severity; Table S12: Comparison of the present study with previous human studies evaluating soluble TLR4 in knee osteoarthritis.

## Abbreviations

The following abbreviations are used in this manuscript:

ACR	American College of Rheumatology
AISI	Aggregate Index of Systemic Inflammation
AUC	Area Under the Curve
BMI	Body Mass Index
CI	Confidence Interval
CRP	C-Reactive Protein
CTX-II	C-Terminal Telopeptide of Type II Collagen
ELISA	Enzyme-Linked Immunosorbent Assay
ESR	Erythrocyte Sedimentation Rate
hsCRP	High-sensitivity C-reactive protein
INF-OA	Inflammatory Osteoarthritis
IRF3	Interferon Regulatory Factor 3
KL	Kellgren–Lawrence
KOFUS	Knee Osteoarthritis Flare-Ups Score
MLR	Monocyte-to-Lymphocyte Ratio
MOAKS	MRI Osteoarthritis Knee Score
MRI	Magnetic Resonance Imaging
NF-κB	Nuclear Factor Kappa B
NLR	Neutrophil-to-Lymphocyte Ratio
non-INF-OA	Non-Inflammatory Osteoarthritis
OA	Osteoarthritis
OR	Odds Ratio
PLR	Platelet-to-Lymphocyte Ratio
ROC	Receiver Operating Characteristic
SII	Systemic Immune-Inflammation Index
SIRI	Systemic Inflammation Response Index
sTLR4	Soluble Toll-Like Receptor 4
sTREM-1	Soluble Triggering Receptor Expressed on Myeloid Cells-1
TLR4	Toll-Like Receptor 4
TREM-1	Triggering Receptor Expressed on Myeloid Cells-1
VAS	Visual Analog Scale
WOMAC	Western Ontario and McMaster Universities Osteoarthritis Index
