# Supplementary material for: Circulating sTLR4 and sTREM-1 in Knee Osteoarthritis: Associations with Study-Specific MRI-Defined Categories and Disease Burden
Source: J Clin Med. 2026 Sep 6;15(17):6902. doi: 10.3390/jcm15176902 (PMC13566080; doi:10.3390/jcm15176902)
Supplement: Supplementary file 1 [file jcm-15-06902-s001.zip › Supplementary Methods.pdf]

Supplementary Methods

Supplementary Methods S1. Trigger-Based Exclusion of Inflammatory and Rheumatologic Mimics

Before KOFUS assessment and MRI-based phenotype assignment, all patients undergoing evaluation for inclusion in the OA cohort were screened for inflammatory, infectious, crystal-induced, and systemic rheumatologic conditions that could produce knee synovitis or alter systemic inflammatory measurements. A sequential trigger-based algorithm was applied. Clinical examination and first-line laboratory screening were performed in all patients, whereas disease-specific serological tests, synovial fluid analysis, and targeted imaging examinations were undertaken only when predefined clinical or laboratory findings raised suspicion of an alternative diagnosis. Serum sTLR4 and sTREM-1 concentrations and blood cell-derived inflammatory indices were not available during this process and were not used to determine eligibility or phenotype assignment.

S1.1. Clinical Screening

A standardized clinical history and musculoskeletal examination were performed to identify features inconsistent with uncomplicated knee OA. Morning stiffness lasting longer than 60 min, persistent swelling of multiple joints, and symmetric small-joint involvement prompted further evaluation for rheumatoid arthritis. Asymmetric oligoarthritis, inflammatory or nocturnal back pain, enthesitis, dactylitis, or uveitis prompted assessment for axial or peripheral spondyloarthritis. Psoriatic skin lesions, nail pitting or dystrophy, dactylitis, and enthesitis raised suspicion of psoriatic arthritis.

Abrupt onset of severe monoarthritis, marked joint swelling or erythema, and the presence of tophi prompted evaluation for crystal-induced arthritis. Fever, constitutional symptoms, marked local warmth, rapidly progressive pain, or a clinically substantial effusion prompted urgent exclusion of septic arthritis. A recent genitourinary or gastrointestinal infection raised suspicion of reactive arthritis, whereas an acute polyarticular presentation accompanied by a viral prodrome or relevant exposure history prompted consideration of viral arthritis.

Raynaud phenomenon, photosensitive or malar rash, oral or nasal ulceration, unexplained cytopenia, urinary abnormalities, serosal symptoms, or other systemic manifestations prompted further assessment for systemic lupus erythematosus or another connective tissue disease.

S1.2. First-Line Laboratory Screening

The first-line laboratory panel included complete blood count, erythrocyte sedimentation rate, C-reactive protein, rheumatoid factor, anti-cyclic citrullinated peptide antibody, antinuclear antibody screening, serum uric acid, complete urinalysis, kidney and liver function tests, and fasting blood glucose.

Laboratory findings were interpreted in conjunction with the clinical presentation. An isolated elevation in ESR or CRP was not considered sufficient for exclusion because low-grade systemic inflammatory abnormalities may occur in OA and other non-rheumatologic

conditions. Similarly, isolated positivity for rheumatoid factor, anti-CCP, or ANA was not considered independently diagnostic of inflammatory arthritis or systemic autoimmune disease. Hyperuricemia alone was not regarded as evidence of gout or another crystal-induced arthropathy. Abnormal findings instead triggered focused clinical reassessment and, when indicated, disease-specific second-line investigations.

S1.3. Disease-Specific Second-Line Evaluation

S1.3.1. Rheumatoid Arthritis

When rheumatoid arthritis was suspected, the evaluation considered the presence and distribution of clinically apparent synovitis, symptom duration, rheumatoid factor and anti-CCP status, and acute-phase reactants. The 2010 ACR/EULAR rheumatoid arthritis classification framework was used to structure the assessment. Targeted radiography or musculoskeletal ultrasonography was performed when required to clarify the presence of inflammatory synovitis or erosive disease. A positive serological test without compatible clinical synovitis was not considered sufficient to establish rheumatoid arthritis or exclude the participant.

S1.3.2. Systemic Lupus Erythematosus and Other Connective Tissue Diseases

When systemic lupus erythematosus was suspected on the basis of mucocutaneous, hematologic, renal, serosal, musculoskeletal, or other systemic findings, additional investigations included anti-double-stranded DNA and anti-Smith antibodies and complement C3 and C4 measurements, as clinically indicated. Urinalysis and kidney function results were reviewed for renal involvement. The 2019 EULAR/ACR classification framework was used as a structured reference during evaluation. ANA positivity alone was not considered sufficient to diagnose systemic lupus erythematosus or exclude a participant.

S1.3.3. Axial and Peripheral Spondyloarthritis

Patients with inflammatory back pain, asymmetric oligoarthritis, enthesitis, dactylitis, uveitis, or other features suggestive of spondyloarthritis underwent focused evaluation. HLA-B27 testing and sacroiliac joint radiography or MRI were obtained when clinically indicated. The Assessment of SpondyloArthritis International Society classification frameworks for axial and peripheral spondyloarthritis were used to organize the evaluation of suspected cases. Imaging abnormalities or HLA-B27 positivity were interpreted in the context of the complete clinical presentation and did not independently determine exclusion.

S1.3.4. Psoriatic Arthritis

Patients with psoriasis, nail dystrophy, dactylitis, enthesitis, or an inflammatory peripheral joint pattern were evaluated for psoriatic arthritis. The Classification Criteria for Psoriatic Arthritis framework was used as a structured reference. Relevant radiographic findings, rheumatoid factor status, and current or previous psoriatic manifestations were considered together with evidence of inflammatory articular disease. No isolated skin, nail, serological, or imaging finding was regarded as sufficient for exclusion in the absence of a clinically coherent diagnosis.

S1.3.5. Gout and Calcium Pyrophosphate Deposition Disease

When gout was suspected, the evaluation incorporated the clinical pattern of acute arthritis, the presence of tophi, serum uric acid concentration, and, when clinically indicated and technically feasible, synovial fluid analysis for monosodium urate crystals. Musculoskeletal ultrasonography or dual-energy computed tomography was used selectively when additional confirmation was required. The 2015 ACR/EULAR gout classification framework was used to structure the assessment.

When calcium pyrophosphate deposition disease was suspected, conventional radiographs were reviewed for chondrocalcinosis, and ultrasonography or synovial fluid crystal analysis was performed when indicated and feasible. The 2023 ACR/EULAR CPPD classification framework was used as a structured reference. Neither hyperuricemia nor chondrocalcinosis alone was considered sufficient to establish a crystal-induced inflammatory arthritis without an appropriate clinical context.

S1.3.6. Septic Arthritis

Patients presenting with an acutely painful, swollen, or markedly warm knee accompanied by fever, constitutional symptoms, or other findings suggestive of infection underwent urgent evaluation for septic arthritis. When clinically indicated, arthrocentesis was performed for synovial fluid leukocyte count and differential, Gram staining, microbiological culture, and crystal analysis. Additional microbiological testing was selected according to the clinical presentation. Participants in whom septic arthritis or another active musculoskeletal infection was identified were excluded.

S1.3.7. Reactive and Viral Arthritis

Reactive arthritis was considered in patients with acute asymmetric arthritis following a recent gastrointestinal or genitourinary infection. Targeted microbiological or serological testing was performed only when supported by the history and clinical examination. Viral arthritis was considered in patients with an acute inflammatory polyarticular presentation, systemic viral symptoms, or a relevant exposure history. Pathogen-specific serological or microbiological testing was undertaken selectively rather than as a universal screening panel. Participants with clinically supported reactive or viral arthritis were excluded.

S1.4. Imaging- and Synovial Fluid-Supported Exclusion

Targeted conventional radiography and musculoskeletal ultrasonography were used when the clinical or laboratory evaluation suggested an inflammatory or crystal-induced mimic. Marginal erosions, erosions accompanied by new bone formation, chondrocalcinosis, gout-compatible erosions, sacroiliac abnormalities, power Doppler-positive synovitis, the ultrasonographic double-contour sign, crystal deposits, tenosynovitis, and enthesitis were considered findings that supported further disease-specific evaluation. No single imaging feature was treated as an automatic diagnosis or exclusion criterion.

Synovial fluid analysis was not routinely performed in every participant. It was reserved for cases in which the clinical presentation raised suspicion of septic or crystal-induced arthritis.

The research MRI examination of the index knee was used for OA phenotype assignment only after inflammatory and rheumatologic mimics had been excluded. The presence of effusion-synovitis or Hoffa-synovitis on the study MRI was therefore not, by itself, interpreted as evidence of systemic inflammatory arthritis.

S1.5. Final Adjudication and Progression to Phenotype Assignment

Exclusion decisions were based on the integrated interpretation of symptoms, physical examination, first-line laboratory results, disease-specific investigations, synovial fluid findings, targeted imaging, and the relevant classification framework. Classification criteria were used to standardize the evaluation of suspected conditions but were not treated as substitutes for clinical diagnosis. Isolated serological, inflammatory, or imaging abnormalities without a compatible clinical syndrome did not automatically result in exclusion.

Participants in whom inflammatory arthritis, systemic rheumatologic disease, crystal-induced inflammatory arthritis, or active joint infection was identified were excluded before OA phenotype assignment. Patients in whom these mimics were not identified subsequently underwent KOFUS assessment. Those with KOFUS scores ≥ 7 were excluded from the final OA analytical cohort because of active OA flare-up, whereas patients with KOFUS scores <7 proceeded to MRI-based INF-OA or non-INF-OA classification. NLR, PLR, MLR, SII, SIRI, AISI, CRP, ESR, sTLR4, and sTREM-1 were not used at any stage of phenotype assignment.

References Cited in Supplementary Methods

Aletaha, D., Neogi, T., Silman, A. J., et al. (2010). 2010 rheumatoid arthritis classification criteria: An American College of Rheumatology/European League Against Rheumatism collaborative initiative. *Arthritis & Rheumatism, 62*(9), 2569–2581. https://doi.org/10.1002/art.27584

Aringer, M., Costenbader, K., Daikh, D., et al. (2019). 2019 European League Against Rheumatism/American College of Rheumatology classification criteria for systemic lupus erythematosus. *Arthritis & Rheumatology, 71*(9), 1400–1412. https://doi.org/10.1002/art.40930

Rudwaleit, M., van der Heijde, D., Landewé, R., et al. (2009). The development of Assessment of SpondyloArthritis International Society classification criteria for axial spondyloarthritis: Part II—Validation and final selection. *Annals of the Rheumatic Diseases, 68*(6), 777–783. https://doi.org/10.1136/ard.2009.108233

Rudwaleit, M., van der Heijde, D., Landewé, R., et al. (2011). The Assessment of SpondyloArthritis International Society classification criteria for peripheral spondyloarthritis and for spondyloarthritis in general. *Annals of the Rheumatic Diseases, 70*(1), 25–31. https://doi.org/10.1136/ard.2010.133645

Taylor, W., Gladman, D., Helliwell, P., Marchesoni, A., Mease, P., & Mielants, H. (2006). Classification criteria for psoriatic arthritis: Development of new criteria from a large international study. *Arthritis & Rheumatism, 54*(8), 2665–2673. https://doi.org/10.1002/art.21972

Neogi, T., Jansen, T. L. T. A., Dalbeth, N., et al. (2015). 2015 gout classification criteria: An American College of Rheumatology/European League Against Rheumatism collaborative initiative. *Arthritis & Rheumatology, 67*(10), 2557–2568. https://doi.org/10.1002/art.39254

Abhishek, A., Tedeschi, S. K., Pascart, T., et al. (2023). The 2023 ACR/EULAR classification criteria for calcium pyrophosphate deposition disease. *Arthritis & Rheumatology, 75*(10), 1703–1713. https://doi.org/10.1002/art.42619
