# Supplementary material for: Circulating sTLR4 and sTREM-1 in Knee Osteoarthritis: Associations with Study-Specific MRI-Defined Categories and Disease Burden
Source: J Clin Med. 2026 Sep 6;15(17):6902. doi: 10.3390/jcm15176902 (PMC13566080; doi:10.3390/jcm15176902)
Supplement: Supplementary file 1 [file jcm-15-06902-s001.zip › Supplementary Results.pdf]

Revised Supplementary Statistical Results

Supplementary Table S1. Transformation rationale and diagnostic checks

Model	Raw-scale diagnostic	Log10-scale diagnostic	Influence / multicollinearity	Decision
sTLR4	Breusch-Pagan p = .032; Shapiro-Wilk p = .628	Breusch-Pagan p = .084; Shapiro-Wilk p = .607	Maximum Cook's D = .113; VIF = 1.11-1.34	Log10 retained for improved homoscedasticity
sTREM-1	Shapiro-Wilk p = .036; Breusch-Pagan p = .068	Shapiro-Wilk p = .199; Breusch-Pagan p = .070	Maximum Cook's D = .112; VIF = 1.11-1.34	Log10 retained for improved residual normality

No observation exceeded Cook's D = 1. Residual and Q-Q plots were also inspected graphically.

Supplementary Table S2. Systemic inflammatory profiles according to study-specific MRI-defined category

Variable	non-INF-OA	INF-OA	U	r_rb	Raw p	Holm-adjusted p
NLR	2.94 [1.05]	3.11 [1.11]	94.0	.161	.473	1.000
PLR	188.17 [38.50]	166.35 [60.50]	103.0	.080	.728	1.000
MLR	0.31 [0.16]	0.36 [0.21]	90.0	.196	.371	1.000
SII	878.67 [342.00]	849.39 [394.00]	110.0	.018	.951	1.000
SIRI	1.59 [0.64]	1.92 [0.83]	81.0	.277	.208	1.000
AISI	469.82 [276.00]	534.99 [250.00]	92.0	.179	.423	1.000
CRP	6.70 [4.93]	6.60 [5.42]	99.5	.112	.618	1.000
ESR	27.50 [18.00]	34.00 [13.00]	51.0	.545	.012	.096

Data are median [interquartile range]. Holm correction was applied across all eight systemic inflammatory measures.

Supplementary Table S3. Exploratory analyses of circulating sTLR4 and sTREM-1 according to MOAKS inflammation severity

Panel A. Severity-stratified comparison within the INF-OA group

Biomarker	MOAKS grade 1 (n = 6), median [IQR]	MOAKS grade 2–3 (n = 10), median [IQR]	Mann–Whitney U	r_rb	Raw p	Holm-adjusted p
sTLR4, ng/mL	5.76 [0.69]	5.98 [1.65]	27.0	0.10	.792	.792
sTREM-1, pg/mL	505.2 [71.6]	449.7 [183.6]	39.0	−0.30	.368	.735

Panel B. Association between MOAKS inflammation severity and circulating biomarkers across the OA cohort (n = 30)

Biomarker	Spearman ϱ	Raw p	Holm-adjusted p
sTLR4	.206	.275	.551
sTREM-1	.023	.905	.905

MRI inflammation severity was defined as the maximum grade across the MOAKS effusion-synovitis and Hoffa-synovitis features for each patient. For Panel A, patients classified as INF-OA were stratified into mild (maximum grade 1) and moderate-to-severe (maximum grade 2–3) inflammation-related MRI findings. Between-group comparisons were performed using exact two-sided Mann–Whitney U tests, with rank-biserial correlation (r_rb) reported as the effect size. For Panel B, the maximum MOAKS inflammation grade was analyzed as an ordinal variable ranging from 0 to 3, and monotonic associations with circulating biomarker concentrations were evaluated using Spearman rank correlations. Holm correction was applied separately across the two biomarker tests within each panel. All analyses were exploratory.

Supplementary Table S4. Associations of BMI with circulating biomarkers and exploratory BMI indirect-effect analyses

Panel A. Correlations between BMI and circulating biomarkers

Cohort	Biomarker	Spearman ϱ	Raw p	Holm-adjusted p
Full sample (n=60)	sTLR4	−.020	.878	.878

Cohort	Biomarker	Spearman ϱ	Raw p	Holm-adjusted p
Full sample (n=60)	sTREM-1	.232	.075	.150
OA cohort (n=30)	sTLR4	−.318	.087	.174
OA cohort (n=30)	sTREM-1	.125	.510	.510

Panel B. Exploratory indirect effects through BMI

Outcome	Group contrast	Indirect effect*	Bootstrap 95% CI
sTLR4	non-INF-OA vs control	−.0212	−.0559 to .0045
sTLR4	INF-OA vs control	−.0179	−.0536 to .0047
sTLR4	INF-OA vs non-INF-OA	.0033	−.0212 to .0295
sTREM-1	non-INF-OA vs control	.0045	−.0219 to .0324
sTREM-1	INF-OA vs control	.0038	−.0189 to .0301
sTREM-1	INF-OA vs non-INF-OA	−.0007	−.0155 to .0139

*Indirect effects are expressed on the log10 biomarker scale. Multicategorical study group was modeled using indicator variables with healthy controls as the reference category. BMI was modeled as the potential intermediate variable, and age and sex were included as covariates in both the BMI and biomarker models. Percentile 95% confidence intervals were obtained from 10,000 stratified bootstrap resamples. Holm correction was applied separately across the two BMI–biomarker correlation tests within the full sample and OA cohort. Because the data are cross-sectional, the indirect-effect analyses should not be interpreted as establishing temporal or causal mediation.

Supplementary Table S5. Unadjusted three-group and All-OA versus healthy control biomarker analyses

Panel A. Unadjusted three-group comparisons

Biomarker	Control	non-INF-OA	INF-OA	χ^2	ε^2	p	Pairwise comparisons
sTLR4, ng/mL	2.89 [1.65]	5.39 [1.95]	5.92 [1.21]	25.7	.436	<.001	Control < non-INF-OA, p = .002; control < INF-OA, p < .001

Biomarker	Control	non-INF-OA	INF-OA	χ²	ε²	p	Pairwise comparisons
sTREM-1, pg/mL	265.85 [101.00]	490.95 [133.45]	489.20 [152.28]	25.3	.429	<.001	Control < both OA groups, p < .001

Panel B. All-OA versus control comparisons

Biomarker	Controls, median [IQR]	All OA, median [IQR]	U	r_rb	p	Adjusted geometric mean ratio (95% CI)	Adjusted p
sTLR4	2.89 [1.65]	5.60 [1.53]	112.5	.750	<.001	1.85 (1.56-2.20)	<.001
sTREM-1	265.85 [100.98]	490.95 [155.60]	111.0	.753	<.001	1.67 (1.43-1.97)	<.001

Panel C. Exploratory OA-versus-control ROC analyses and internal validation

Biomarker	Apparent AUC	Bootstrap 95% CI	LOOCV AUC	Exploratory Youden threshold	Sensitivity	Specificity	Youden J
sTLR4	.875	.776–.957	.846	≥4.51 ng/mL	83.3%	83.3%	.667
sTREM-1	.877	.774–.961	.850	≥385.2 pg/mL	83.3%	83.3%	.667

Apparent AUC confidence intervals were estimated using 10,000 stratified bootstrap resamples. LOOCV AUCs were calculated from pooled out-of-fold predicted probabilities obtained from single-biomarker logistic regression models, with each participant omitted once. Youden thresholds were selected from the full sample and are therefore sample-specific exploratory estimates. Sensitivity, specificity, and Youden J correspond to these apparent-data thresholds. Neither the thresholds nor the associated operating characteristics should be interpreted as externally validated diagnostic or screening performance.

All-OA adjusted models included age, sex, and BMI.

Supplementary Table S6. Exploratory discrimination between the study-specific MRI-defined OA categories

Panel A. Binary logistic regression

Model	Predictor	OR	95% CI	p
Individual biomarker model	sTLR4, per 1 ng/mL	1.28	0.76-2.17	.357
Individual biomarker model	sTREM-1, per 1 pg/mL	1.003	0.996-1.010	.446
Combined biomarker model	sTLR4 + sTREM-1	LR $\chi^2(2) = 1.40$	—	.497

Panel B. ROC analyses

Marker/model	AUC	95% CI	p
sTLR4	.603	.385-.821	.356
sTREM-1	.442	.228-.656	.596
Combined model-derived probability	.612	.395-.828	.312

Supplementary Table S7. Distributional Characteristics of Serum sTLR4 and sTREM-1

Biomarker	Group	n	Mean	SD	Variance	Median	IQR	Minimum	Maximum
sTLR4	Control	30	3.31	1.23	1.52	2.89	1.65	1.86	6.68
sTLR4	non-INF-OA	14	5.27	1.76	3.09	5.39	1.95	2.37	8.68
sTLR4	INF-OA	16	5.76	1.12	1.26	5.92	1.21	3.08	7.66
sTLR4	All OA	30	5.53	1.45	2.10	5.60	1.53	2.37	8.68
sTREM-1	Control	30	304.17	114.09	13016.22	265.85	100.98	204.20	609.40
sTREM-1	non-INF-OA	14	468.53	113.47	12874.73	490.95	133.45	280.00	635.90
sTREM-1	INF-OA	16	500.45	118.97	14154.12	489.20	152.28	300.70	733.40

Biomarker	Group	n	Mean	SD	Variance	Median	IQR	Minimum	Maximum
sTREM-1	All OA	30	485.55	115.56	13354.88	490.95	155.60	280.00	733.40

Supplementary Table S8. Associations of circulating sTLR4 and sTREM-1 with systemic inflammatory, clinical, and radiographic measures among patients with OA

Variable	sTLR4, ϱ	Raw p	Holm-adjusted p	sTREM-1, ϱ	Raw p	Holm-adjusted p
Systemic inflammatory measures						
CRP	.480	.007	.109	.461	.010	.145
ESR	.591	<.001	.009	.181	.339	1.000
NLR	−.059	.759	1.000	.011	.954	1.000
PLR	−.204	.280	1.000	−.087	.647	1.000
MLR	−.087	.648	1.000	−.294	.115	1.000
SII	−.163	.391	1.000	.019	.922	1.000
SIRI	−.087	.646	1.000	−.206	.274	1.000
AISI	−.215	.255	1.000	−.146	.443	1.000
Clinical and radiographic measures						
VAS	.442	.015	.087	.473	.008	.071
WOMAC pain	.416	.022	.111	.314	.091	.273
WOMAC stiffness	.538	.002	.024	.534	.002	.024
WOMAC function	.569	.001	.012	.366	.047	.186

Variable	sTLR4, ϱ	Raw p	Holm-adjusted p	sTREM-1, ϱ	Raw p	Holm-adjusted p
WOMAC total	.669	<.001	<.001	.475	.008	.071
Kellgren-Lawrence grade	.672	<.001	<.001	.455	.012	.081
OA disease duration	−.229	.224	.447	.076	.691	.691

Holm correction was applied separately to the systemic inflammatory and clinical/radiographic families.

Supplementary Table S9. Alternative scaling and bootstrap sensitivity analyses for sTLR4

Panel A. Raw-unit and standardized ordinal estimates

Model	Scaling	OR	95% CI	p
Primary	Per 1 ng/mL	7.12	2.10-24.14	.002
Primary	Per 1-SD increase	17.18	2.93-100.60	.002
Sensitivity (+ disease duration)	Per 1 ng/mL	6.58	2.05-21.09	.002
Sensitivity (+ disease duration)	Per 1-SD increase	15.30	2.83-82.71	.002

Panel B. Bootstrap confidence intervals

Analysis	Point estimate	Method	Bootstrap 95% CI	Interpretation
sTLR4 → WOMAC total	B = 4.99	10,000 case resamples	2.31-7.46	Stable
sTLR4 → higher KL grade, per 1 SD	OR = 17.18	10,000 case resamples	3.87-93,110	Highly unstable/right-skewed

Analysis	Point estimate	Method	Bootstrap 95% CI	Interpretation
sTLR4 ROC: OA vs control	AUC = .875	10,000 stratified resamples	.776-.957	Stable
sTREM-1 ROC: OA vs control	AUC = .877	10,000 stratified resamples	.774-.961	Stable

Only 9,396 of 10,000 ordinal-model resamples yielded finite estimates. The model-based Wald CI was therefore retained as the primary interval.

Supplementary Table S10. Exploratory interaction analyses of the association between circulating sTLR4 and WOMAC total score

Effect modifier	Interaction term	B	95% CI	Raw p	Holm-adjusted p
BMI	sTLR4 × BMI	0.101	−0.664 to 0.866	.788	1.000
Age	sTLR4 × age	−0.042	−0.581 to 0.497	.873	1.000
MRI category	sTLR4 × MRI category	−3.853	−8.821 to 1.115	.122	.367

WOMAC total score was the dependent variable in all models. Interaction terms were examined in separate exploratory linear regression models to limit overparameterization in the 30-patient OA cohort. Continuous predictors were mean-centered before construction of product terms. The BMI interaction model included sTLR4, BMI, age, and the sTLR4 × BMI term; the age interaction model included sTLR4, age, BMI, and the sTLR4 × age term; and the MRI category interaction model included sTLR4, age, BMI, MRI category, and the sTLR4 × MRI category term. The study-specific MRI category was coded as non-INF-OA = 0 and INF-OA = 1. Holm correction was applied across the three interaction tests. All analyses were exploratory.

Supplementary Table S11. Sensitivity analyses of the association between circulating sTLR4 and Kellgren–Lawrence severity

Panel A. Distribution of sTLR4 according to KL grade

KL grade	n	sTLR4, median [IQR], ng/mL
KL2	9	4.24 [3.08–5.20]
KL3	16	5.85 [5.40–6.35]
KL4	5	7.60 [6.69–7.66]

Panel B. Logistic regression sensitivity analyses

Comparison	n	OR per 1 ng/mL sTLR4	95% CI	Raw p	Holm-adjusted p
KL3–4 vs KL2	30	6.21	1.47–26.27	.013	.026
KL4 vs KL3	21	3.62	1.01–12.91	.047	.047

The OA cohort contained no KL grade 1 observations. Accordingly, a separate ordinal regression within the mild KL1–2 stratum was not estimable because all patients in this stratum had KL grade 2. To address potential instability related to sparse KL categories, KL severity was first dichotomized as KL2 versus KL3–4. A second exploratory model compared KL4 with KL3 within the moderate-to-severe subgroup. Models were intentionally unadjusted and parsimonious because of the small grade-specific sample sizes. Odds ratios are reported per 1 ng/mL increase in sTLR4. Holm correction was applied across the two sensitivity tests.

Supplementary Table S12. Comparison of the present study with previous human studies evaluating soluble TLR4 in knee osteoarthritis

Study	Design and sample	sTLR4 compartment	OA / phenotype definition	Main sTLR4-relevant endpoints	Main finding relevant to sTLR4
Barreto et al., 2017	Cross-sectional tissue and synovial-fluid study; sTLR4 analysis: 10 knee OA, 10 healthy knee controls, 10 CMC-I OA	Synovial fluid	Knee OA samples obtained from patients undergoing TKA for severe OA; no MRI inflammatory phenotype	Synovial-fluid sTLR4; tissue TLR expression across joints	sTLR4 was detectable in OA synovial fluid and was higher in knee OA than in healthy knees; levels were highest in CMC-I OA
Huang et al., 2018	Longitudinal biomarker analysis of DOXY trial; n=431	EDTA plasma	Obese women aged 45–64 years; unilateral radiographic knee OA; KL2–3 index knee and KL0–1 contralateral knee; no MRI inflammatory phenotype	Baseline/time-integrated sTLR4; ΔuCTX-II at 18 months; JSW/JSN	Baseline and time-integrated sTLR4 were associated with increased uCTX-II;

Study	Design and sample	sTLR4 compartment	OA / phenotype definition	Main sTLR4-relevant endpoints	Main finding relevant to sTLR4
				progression at 16 months	sTLR4 was not the principal signal for radiographic JSW/JSN progression
Present study	Prospective cross-sectional biomarker study; 30 OA + 30 healthy controls; OA: 14 non-INF-OA and 16 INF-OA	Serum	OA confirmed clinically/radiographically; MRI operational phenotype based on MOAKS effusion-synovitis and/or Hoffa-synovitis ≥1 for INF-OA	MRI phenotype specificity; OA-control differences; WOMAC burden; KL severity	sTLR4 did not distinguish INF-OA from non-INF-OA but was elevated in OA vs controls and associated with WOMAC-defined and radiographic disease burden

Abbreviations: CMC-I, first carpometacarpal joint; INF-OA, inflammatory osteoarthritis; JSN, joint-space narrowing; JSW, joint-space width; KL, Kellgren–Lawrence; MOAKS, MRI Osteoarthritis Knee Score; OA, osteoarthritis; sTLR4, soluble Toll-like receptor 4; TKA, total knee arthroplasty; uCTX-II, urinary C-telopeptide of type II collagen. The studies differ substantially in biological compartment, study population, OA definition, and outcome structure; direct comparison of absolute sTLR4 concentrations across studies is therefore not appropriate.
